# B.1.1.7 (Alpha) variant is the most antigenic compared to Wuhan strain, B.1.351, B.1.1.28/triple mutant and B.1.429 variants

**DOI:** 10.3389/fmicb.2022.895695

**Published:** 2022-08-12

**Authors:** Manojit Bhattacharya, Ashish Ranjan Sharma, Bidyut Mallick, Sang-Soo Lee, Eun-Min Seo, Chiranjib Chakraborty

**Affiliations:** ^1^Department of Zoology, Fakir Mohan University, Balasore, Odisha, India; ^2^Institute for Skeletal Aging and Orthopedic Surgery, Hallym University-Chuncheon Sacred Heart Hospital, Chuncheon, Gangwon-do, South Korea; ^3^Department of Applied Science, Galgotias College of Engineering and Technology, Greater Noida, Uttar Pradesh, India; ^4^Department of Biotechnology, School of Life Science and Biotechnology, Adamas University, Kolkata, West Bengal, India

**Keywords:** emerging variants, CTL epitopes, B.1.1.7, antigenicity, cluster, phage-displayed peptides, CD spectra, SAg-like region

## Abstract

The rapid spread of the SARS-CoV-2 virus and its variants has created a catastrophic impact worldwide. Several variants have emerged, including B.1.351 (Beta), B.1.1.28/triple mutant (P.1), B.1.1.7 (Alpha), and B.1.429 (Epsilon). We performed comparative and comprehensive antigenicity mapping of the total S-glycoprotein using the Wuhan strain and the other variants and identified 9-mer, 15-mer, and 20-mer CTL epitopes through *in silico* analysis. The study found that 9-mer CTL epitope regions in the B.1.1.7 variant had the highest antigenicity and an average of the three epitope types. Cluster analysis of the 9-mer CTL epitopes depicted one significant cluster at the 70% level with two nodes (KGFNCYFPL and EGFNCYFPL). The phage-displayed peptides showed mimic 9-mer CTL epitopes with three clusters. CD spectra analysis showed the same band pattern of S-glycoprotein of Wuhan strain and all variants other than B.1.429. The developed 3D model of the superantigen (SAg)-like regions found an interaction pattern with the human TCR, indicating that the SAg-like component might interact with the TCR beta chain. The present study identified another partial SAg-like region (ANQFNSAIGKI) from the S-glycoprotein. Future research should examine the molecular mechanism of antigen processing for CD8^+^ T cells, especially all the variants’ antigens of S-glycoprotein.

## Introduction

The COVID-19 has affected the global population, and a surge of the SARS-CoV-2 has been noted in many countries (Chakraborty et al., 2020; Dinesh et al., 2020; Li and Liu, 2020). Due to this COVID-19 wave, high peaks of daily infection and death occurred in these countries. One of the most significant causes of surges in different countries is the emergence of new variants, developing because of mutations (Brüssow, 2021).

**Figure 1:**
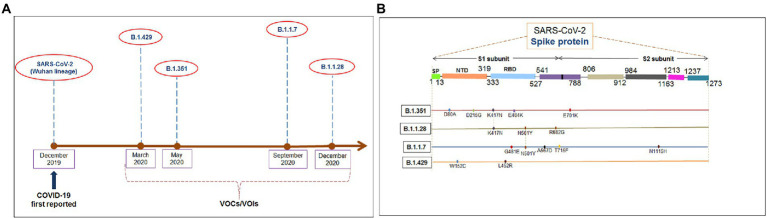
The figure depicted the timeline and mutations of newly significant emerging variants and Wuhan strain. **(A)** Timeline showing the origin time of some newly significant emerging variants (B.1.351, B.1.1.28/triple mutant, B.1.1.7, and B.1.429) and Wuhan strain. **(B)** The schematic diagram shows the significant mutations present in the CTL antigenic regions of newly emerging SARS-CoV-2 variants S-protein.

Several significant variants of concern/interest (VOCs/VOIs) of this virus [B.1.351, (Beta), B.1.1.28/triple mutant (P.1), B.1.1.7 (Alpha), and B.1.429 (Epsilon)] have been determined in South Africa, Brazil, United Kingdom, and the United States and have mushroomed to many other countries (Chakraborty et al., 2021a,b; Focosi et al., 2021; Harvey et al., 2021). These newly evolved variants with significant mutations have developed from the original Wuhan strain in different regions worldwide. The ongoing evolution of the SARS-CoV-2 virus is due to the region-wise adaptive changes and multiple mutations during the evolutionary process (Chakraborty et al., 2021c; Rochman et al., 2021). Diverse variants of this virus have developed over time due to the several mutations (Figure 1A). However, four significant variants (B.1.351, B.1.1.28/triple mutant, B.1.1.7, and B.1.429) have been identified in different regions worldwide. All four variants have mutations within the spike (S) protein, which change the attachment site of the virus, human cell entry, and strong binding affinity with VOC/VOI and receptor compared to the Wuhan strain. The UK variant (B.1.1.7) steadily surged in just a few months and is now found in at least 114 countries worldwide (Davies et al., 2021). Several significant mutations have been identified in the B.1.1.7 lineage. One significant mutation (N501Y) was observed in the viral S-glycoprotein of the receptor-binding domain (RBD). Similarly, four mutations were observed in the S1-domain (del144Y, del69–70 HV, D614G, and A570D), and four mutations were noted in the S2 domain (P681H, S982A, T761I, and D1118H) (Li et al., 2021; Meng et al., 2021; Shen et al., 2021). The B.1.351 lineage consists of three significant mutations in the RBD region of the S-glycoprotein. These mutations are noted as K417N, E484K, and N501Y. Five important mutations have been observed in the N-terminal domain: R246I, D215G, L18F, D80A, and a deletion mutation at amino acid positions 242–244. Another mutation (A701V) in the S2 subunit has also been observed (Cele et al., 2021; Wang et al., 2021b). The P.1 (B.1.1.28.1) lineage has three significant mutations (K417T, E484K, and N501Y) in the RBD region of the S-glycoprotein, and eight other significant amino acid changes have been found (D138Y, T20N, L18F, R190S, P26S, T1027I, H655Y, and V1176F). The B.1.429 lineage has four mutations, two mutations (L452R and D614G) reside within the RBD, and two mutations (S13I and W152C) are found in the NTD (N-terminal domain) segment of the S-glycoprotein. These mutational changes exhibit augmented neutralization resistance (Faria et al., 2021; Wang et al., 2021a). The present study has explored the antigenicity of B.1.1.28/triple mutant along with two VOCs (B.1.1.7 and B.1.351). Similarly, several mutations have been noted in the S-glycoprotein of these variants (Naveca et al., 2021).

Antigenicity mapping is a powerful technique for understanding the antigenic properties of any virus and can be analyzed using epitope mapping (Sun et al., 2013; Anderson et al., 2018). Antigenicity mapping can help to understand different characteristics such as antigenic sites and immune escape mechanisms (Peacock et al., 2016). CD8^+^ T cells epitopes are also called cytotoxic T lymphocytes (CTL) epitopes and are one of the critical determinants for understanding the CD8^+^ T cell response and antigenic properties (Feliu et al., 2013; Tscharke et al., 2015). Recently, Gangaev et al. (2021) attempted to map the immunodominant and immunogenic epitopes of this virus to illustrate the role of CD8^+^ T cells in host defense (Gangaev et al., 2021). Understanding CTL epitopes with antigenic properties can assist in vaccine design (Wong et al., 2019; Bhattacharya et al., 2020a,b, 2021). Therefore, CTL epitope mapping plays a vital role in determining the antigenicity of a pathogen. Researchers have examined 9-mer, 15-mer, and 20-mer CTL epitopes to understand the antigenicity of pathogens and designed vaccines against these pathogens (Doi et al., 2009; Bhatnager et al., 2020; Rencilin et al., 2021; Shey et al., 2021). The 9-mer CTL epitopes determine the viral antigenicity (Raza et al., 2021). Several researchers have used 9-mer CTL epitopes to assess the antigenic peptides from S-glycoprotein, and this 9-mer CTL epitope has been used for vaccine development against SARS CoV-2 (Kar et al., 2020; Rencilin et al., 2021).

Epitope clustering strategies might help capture the diversity of pathogens’ epitopes and understand highly similar or overlapping sequences. Previous studies have examined CTL epitope clustering for different pathogens (Liu et al., 2010; Rist et al., 2015; Vujovic et al., 2020). In the present study, the epitope clustering method was used to understand the clustering of CTL epitopes of the S-glycoprotein of the Wuhan strain (wild type) and mutant variants of SARS-CoV-2. Circular dichroism (CD) spectra provide information on the molecular structure of proteins at a quantitative level (Micsonai et al., 2015). The CD spectra can also provide information on the molecular structure of a mutant protein (Huai et al., 2021). We analyzed the CD spectra of the Wuhan strain and mutant variants of the S-glycoprotein of this virus.

In patients with severe cases, toxic shock syndrome has been observed, which causes hyperinflammation. Cheng et al. (2020) observed a superantigenic insert, which is a unique S-glycoprotein, in SARS-CoV-2, and concluded that the insert might be responsible for causing hyper inflammation in patients with severe COVID-19 (Cheng et al., 2020). SAgs can excessively activate the immune system. Previous studies have identified SAgs from viruses (Lafon, 2000). A SAg-like region (PRRA) was recently detected in this virus S-glycoprotein of the Wuhan strain (Cheng et al., 2020). We attempted to analyze the superantigenic insert in the Wuhan strain and mutant variants further.

In this study, our objective was to perform a comprehensive *in silico* analysis of the total S-glycoprotein of the Wuhan strain, B.1.351, B.1.1.28/triple mutant, B.1.1.7, and B.1.429 variants to understand the antigenicity using the CTL epitopes or SAg-like region of S-glycoprotein and the interaction between the SAg-like area within the and the T-cell antigen receptor (TCR).

## Materials and methods

### Data acquisition of the S-glycoprotein of the Wuhan strain and B.1.351, B.1.1.28/triple mutant, B.1.1.7, and B.1.429 variants

The sequences of amino acids within the S protein of the Wuhan strain and the four significant mutant variants were retrieved from the NCBI/PDB (protein database) in FASTA format. The accession numbers of the Wuhan strain and the B.1.351, B.1.1.28/triple mutant, B.1.1.7, and B.1.429 variants are QHR63290.2, 7LYL_A, 7LWW_A, 7LWV_A, and 7N8H_A, respectively. Similarly, we collected PDB files from the PDB database for the Wuhan strain and the B.1.351, B.1.1.28/triple mutant, B.1.1.7, and B.1.429 variants, 6VXX 7LYL, 7LWW, 7LWV, and 7N8H, respectively. We started our work with the PDB immediately at that time of deposition of the structure for 7LWW. We found that the 7LWW was mentioned as structure S-glycoprotein of the B.1.1.28 in the PDB database.

Afterward, we found that the PDB is mentioned as a triple mutant in some pieces of literature. In this study, we have used both the two parameters (“B.1.1.28” and “triple mutant”) at the same time for describing the 7LWW and mentioning it as B.1.1.28/triple mutant.

### Identification of 9-mer, 15-mer, and 20-mer CTL epitopes of total S-glycoprotein and RBD region of S-glycoprotein of the Wuhan strain and the B.1.351, B.1.1.28/triple mutant, B.1.1.7, and B.1.429 variants

We identified the 9-mer, 15-mer, and 20-mer CTL epitopes of S-glycoprotein from the Wuhan strain and all four variants (B.1.351, B.1.1.28/triple mutant, B.1.1.7, and B.1.429) and analyzed their antigenic sequences. To identify any promising MHC-I (Major Histocompatibility Complex class I) binding epitopes, we used ProPred-I, which is an online web server for predicting 9-mer, 15-mer, and 20-mer peptide binding to MHC-I alleles (Singh and Raghava, 2003). Finally, we calculated the average number of epitopes in the five variants.

### Antigenicity predictions of the identified epitopes of the S-glycoprotein of the Wuhan strain and the B.1.351, B.1.1.28/triple mutant, B.1.1.7, and B.1.429 variants

In the present study, we employed the VaxiJen server, which uses a unique algorithm comprising an auto cross-covariance-based machine-learning server and an alignment-based prediction process (Doytchinova and Flower, 2007). This server uses a default parameter and threshold to calculate the antigenicity of pathogens. Here, we applied 0.4 as the threshold value for the viral peptide.

### Comprehensive and comparative analysis of significant 9-mer CTL epitopic landscape of S-glycoprotein of the Wuhan strain and the B.1.351, B.1.1.28/triple mutant, B.1.1.7, and B.1.429 variants

We performed a comprehensive comparative analysis of the Wuhan strain’s significant 9-mer CTL epitopic landscape and significant VOCs/VOI/others. We attempted to determine the different essential mutations present in the 9-mer CTL epitopes in the B.1.351, B.1.1.28/triple mutant, B.1.1.7, and B.1.429 variants. Finally, we hypothesized that the epitopes mutated from the Wuhan strain evolved into different significant VOCs/VOI/others.

### MSA using the identified CTL epitopes (9-mer, 15-mer, and 20-mer) of the S-glycoprotein of the Wuhan strain and B.1.351, B.1.1.28/triple mutant, B.1.1.7, and B.1.429 variants

The 9-mer, 15-mer, and 20-mer CTL epitopes of the S-glycoprotein of the Wuhan strain and the B.1.351, B.1.1.28/triple mutant, B.1.1.7, and B.1.429 variants were used to perform MSA. MSA was used to undertake sequence alignment with Clustal Omega (Sievers and Higgins, 2018, 2021). The tool applied the HHalign technique to align the landscape of HMMs (Hidden Markov models).

### Cluster analysis of CTL epitopes (9-mer, 15-mer, and 20-mer) of S-glycoprotein of the Wuhan strain and the B.1.351, B.1.1.28/triple mutant, B.1.1.7, and B.1.429 variants

The clustering of peptides is a well-known problem in biological science. The analysis of the clustering of epitopes informs us of the epitope homology. It also helps us to generate a precise consensus sequence, which is essential to understanding a group of sequences sharing a defined level of identity. We performed cluster analysis at the different threshold levels (10 to 80%).

The clustering tool resolves the similarity between peptide sequences in separate and specific clusters. It is also ideal for analyzing sequences that may cluster together, even though they are derived explicitly from distinct antigenic sequences. It also specifies and identifies regions of homology between unrelated peptide sequences linked with several critical biological phenomena (allergen cross-reactivity, molecular mimicry, modulation of adaptive immune responses). Researchers also identified the epitopes in a large-scale screen of overlapping peptides that frequently share substantial sequence similarities, usually complicating the analysis of epitope-related data (Stufano et al., 2010). Therefore, clustering algorithms are often used to simplify these analyses. However, existing approaches are commonly inadequate in their capacity to define the biologically meaningful epitope clusters in the advanced framework of the immune response. Therefore, this algorithm-based IEDB epitope cluster analysis tool is applied to generate epitope clusters based on consensus or representative sequences (Vita et al., 2015). This specialized tool also allows the researchers to cluster the target peptide sequences based on a specified level of identity by selecting among changed method options (Dhanda et al., 2018). From a computational standpoint and advanced phase of immunoinformatics, the clustering tool is flexible enough to consider diverse strategies to address different immunological queries using B cell and T cell epitopes (Vivona et al., 2008).

We performed cluster analysis of the CTL epitopes (9-mer, 15-mer, and 20-mer) of S-glycoprotein of the Wuhan strain and the B.1.351, B.1.1.28/triple mutant, B.1.1.7, and B.1.429 variants. We used the IEDB epitope cluster analysis tool (Zhang et al., 2008; Dhanda et al., 2018). This tool graph visualizes the generated network using Python networks.

### Exploration of the CD spectra and different components of the secondary structure of S-glycoprotein of the Wuhan strain and the B.1.351, B.1.1.28/triple mutant, B.1.1.7, and B.1.429 variants

CD spectroscopy is a significant parameter for understanding the protein structure. A CD spectrum is an essential component that can help rapidly determine the secondary structure of proteins (Greenfield, 2006). We can understand different secondary structure elements using the CD spectra *via* MD simulations (Rogers et al., 2019; Drew and Janes, 2020). The PDBMD2CD is a server that can generate CD spectra using PDB files. Using this server, we analyzed the CD spectra of the S-glycoprotein of the Wuhan strain and significant VOCs/VOIs (Drew and Janes, 2020). The analysis illustrated information on the wavelength of the CD signal. The server produces a result with an RMSD smaller or equivalent to 0.5, the utmost away from the investigational spectrum. We used this server to analyze the secondary structural features such as the helix, antiparallel sheet, parallel sheet, and turn of the S-glycoprotein of the Wuhan strain and the B.1.351, B.1.1.28/triple mutant, B.1.1.7, and B.1.429 variants.

### Tertiary or 3D model generation of the 9-mer CTL epitopic regions

We developed the tertiary structure of 9-mer CTL epitopes from the S-glycoprotein of the Wuhan strain and significant VOCs/VOIs/others using the DISTILL 2.0 server (Baú et al., 2006). This tool adopts an algorithm based on recursive neural network architectures. The algorithm can predict through a specific architecture of a neural network (single-or dual-layer) and directs an acyclic graph (Li et al., 2019). Different epitopes were marked in the tertiary structure, generated from the PDB file. The 3D structure of the PDB was developed using chimera visualization software (Pettersen et al., 2004).

### Phage-displayed peptides that mimic SARS-CoV-2 9-mer CTL epitopic peptides

A peptide library for phage-displayed was applied for epitope mapping (Wu et al., 2016). We analyzed the SARS-CoV-2 9-mer CTL epitopic peptides that mimic phage-displayed peptides’ epitopes. We used a routine technique to map the location of the epitope using a phage-display library. We used the Pepitope server to perform this analysis, which applied the PepSurf algorithm. This algorithm is based on a specific amino acid similarity matrix (Mayrose et al., 2007).

### Identification of the SAg-like region from the S-glycoprotein of SARS-CoV-2 of the Wuhan strain and B.1.351, B.1.1.28/triple mutant, B.1.1.7, and B.1.429 variants, and their tertiary or 3D model generation

We have identified the SAg-like region from the S-glycoprotein of SARS-CoV-2 of the Wuhan strain and B.1.351, B.1.1.28/triple mutant, B.1.1.7, and B.1.429 variants. We developed a 3D structure model using the same SAg-like region from the S-glycoprotein of Wuhan strain and the B.1.351, B.1.1.28/triple mutant, B.1.1.7, and B.1.429 variants using the DISTILL 2.0 server (Baú et al., 2006).

### Interaction between SAg-like part of S-glycoprotein of SARS-CoV-2 and TCR

A 3D structural model of the SAg-like region of the S-glycoprotein of this virus and TCR interaction was illustrated with the HADDOCK server (PDB ID: 4WW1; De Vries et al., 2010), which was used for protein–protein docking. The docking analysis was further validated using the ClusPro server (Kozakov et al., 2017).

### Identification of the partial SAg-like part of this virus S-glycoprotein of the Wuhan strain and the B.1.351, B.1.1.28/triple mutant, B.1.1.7, and B.1.429 variants

The SAgs used in the present study were: α-cobra toxin (*Naja naja*), α-bungarotoxin, rabies virus G protein (189–199), α-cobra toxin (*Naja kaouthia*), and HIV-1 gp120 (164–174). We utilized the entire sequence of the S-glycoprotein of the Wuhan strain and B.1.351, B.1.1.28/triple mutant, B.1.1.7, and B.1.429 variants during the present study. We found another SAg-like part from the S-glycoprotein of the Wuhan strain and B.1.351, B.1.1.28/triple mutant, B.1.1.7, and B.1.429 variants. The peptide sequence of the SAg-like part of all these SARS-CoV-2 virus sequences (both wild and mutant type) was used for cluster formation.

**Figure 2:**
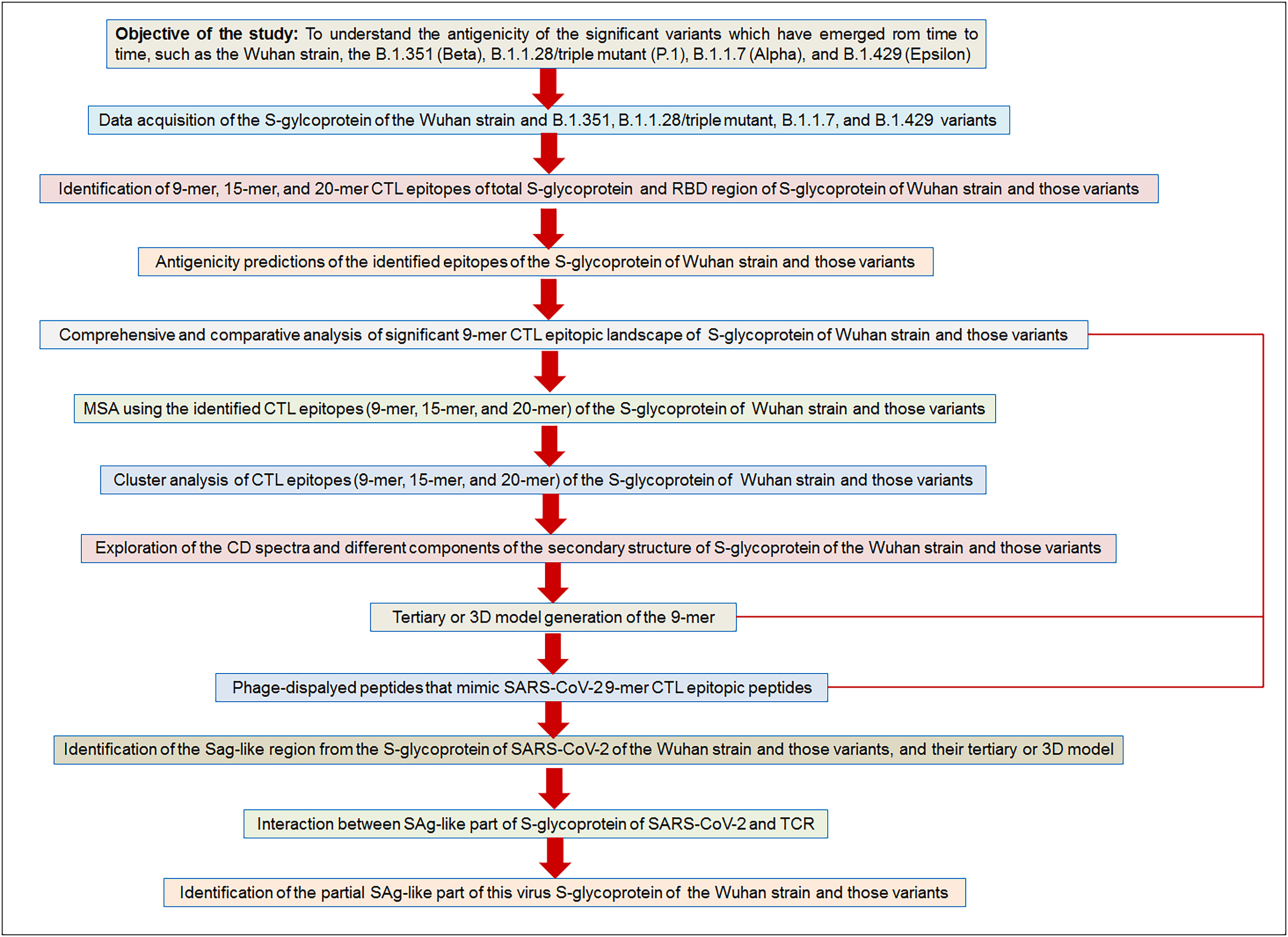
The flow diagram illustrates our used methodologies to test the objective of the study hypothesis. Our aim/objective of this research is to understand the antigenicity of the significant variants which have emerged from time to time, such as the Wuhan strain, the B.1.351 (Beta), B.1.1.28/triple mutant (P.1), B.1.1.7 (Alpha), and B.1.429 (Epsilon).

Finally, we have depicted a flowchart of the materials and methods to provide a bird’s eye view of the current study (Figure 2).

## Results

### Total number of CTL epitopes (9-mer, 15-mer, and 20-mer) in the S-glycoprotein of the Wuhan strain and B.1.351, B.1.1.28/triple mutant, B.1.1.7, and B.1.429 variants

**Figure 3:**
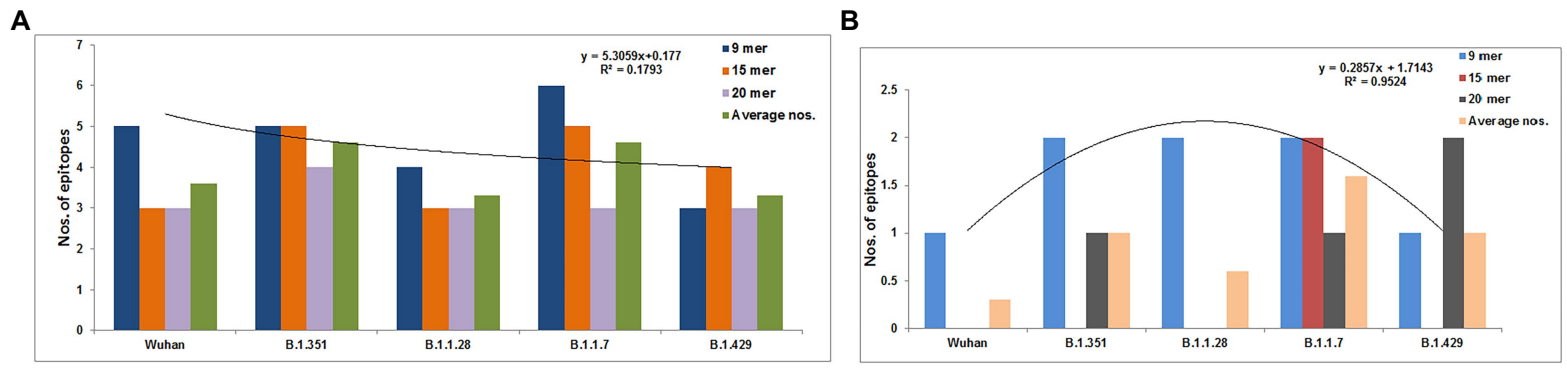
Comparison of the number of the 9 mer, 15 mer, 20 mer CTL antigenic epitopes of total S-glycoprotein and RBD region of S-glycoprotein of Wuhan strain and B.1.351, B.1.1.28/triple mutant, B.1.1.7, and B.1.429 variant. **(A)** Comparison of the number of the 9 mer, 15 mer, 20 mer, and an average number of CTL antigenic epitopes of total S-glycoprotein. **(B)** Comparison of the number of 9 mer, 15 mer, 20 mer, and the average number of CTL antigenic epitopes of RBD region of S-glycoprotein. We develop the polynomial (order 2) relationships for the two graphs (**A**; *R*^2^ = 0.1793 and **B**; *R*^2^ = 0.9524).

We identified 9-mer, 15-mer, and 20-mer CTL epitopes in the S-glycoprotein of the Wuhan strain and B.1.351, B.1.1.28/triple mutant, B.1.1.7, and B.1.429 variants. We found that B.1.1.7 contained a maximum number of 9-mer CTL epitopes (approximately six), whereas the B.1.429 variant had a minimum number of 9-mer CTL epitopes (approximately three; Figure 3A). We noted a maximum number of 15-mer CTL epitopes in the S-glycoprotein in B.1.1.7 and B.1.351 (approximately five), whereas the Wuhan strain and B.1.1.28/triple mutant variant contained a minimum number of 15-mer CTL epitopes in the S-glycoprotein (approximately three; Figure 3A; Supplementary Table S1). However, the 20-mer CTL epitopes in the S-glycoprotein showed slightly different results. We found a maximum number of 20-mer CTL epitopes in the S-glycoprotein in B.1.351 (approximately four). In contrast, the Wuhan strain and the B.1.1.28/triple mutant, B.1.1.7, and B.1.429 variants contained a minimum number of 20-mer CTL epitopes in the S-glycoprotein (approximately three; Figure 3A; Supplementary Table S2). Calculating the average number of epitopes found that the average number of epitopes was the same in B.1.1.7 and B.1.351 (Figure 3A).

### Total number of CTL epitopes (9-mer, 15-mer, and 20-mer) in the RBD region of S-glycoprotein of the Wuhan strain and the B.1.351, B.1.1.28/triple mutant, B.1.1.7, and B.1.429 variants

We identified 9-mer, 15-mer, and 20-mer CTL epitopes in the RBD region of the S-glycoprotein of the Wuhan strain and the B.1.351, B.1.1.28/triple mutant, B.1.1.7, and B.1.429 variants. We found that the B.1.1.7, B.1.351, and B.1.1.28/triple mutant variants contained a maximum number of 9-mer CTL epitopes (approximately two). In contrast, the Wuhan strain and B.1.429 variant had a minimum number of 9-mer CTL epitopes (approximately one; Figure 3B).

We noted a maximum number of 15-mer CTL epitopes in the RBD region of the S-glycoprotein in the B.1.1.7 variant (approximately two). In contrast, the Wuhan strain and B.1.351, B.1.1.28/triple mutant, and B.1.429 variants contained no 15-mer CTL epitopes (Figure 3B).

However, the 20-mer CTL epitopes in the RBD region showed slightly different results. We found one 20-mer CTL epitope in the RBD region in B.1.351 and B.1.1.7. The other two variants (Wuhan strain and the B.1.1.28/triple mutant) contained no 20-mer CTL epitopes in the RBD region of this virus (Figure 3B).

By calculating the average number of epitopes in the RBD region, we found that highest average number of epitopes was contained in the B.1.1.7 variant (Figure 3A).

### Comprehensive analysis of significant 9-mer CTL epitopic landscape of S-glycoprotein of the Wuhan strain and the B.1.351, B.1.1.28/triple mutant, B.1.1.7, and B.1.429 variants

We performed comparative antigenicity mapping of the S-glycoprotein on the Wuhan strain and B.1.351, B.1.1.28/triple mutant, B.1.1.7, and B.1.429 variants, with 23 epitopes identified. Four epitopes were non-antigenic based on their VaxiJen score (> 0.4). The significant mutations in the variants containing the 9-mer epitope played a crucial role in viral antigenicity (Figure 1B). Five 9-mer antigenic CTL epitopes were identified from the S-glycoprotein of the Wuhan strain. Similarly, the S-glycoprotein of B.1.351 contained five 9-mer CTL antigenic epitopes covering the mutagenic regions. Among these five epitopes, three were highly antigenic, as predicted by the VaxiJen server. The S-glycoprotein of the B.1.1.28/triple mutant variant contained four 9-mer CTL antigenic epitopes. We found the highest number of 9-mer CTL antigenic epitopes in the B.1.1.7 variant (approximately six), with all six antigenic epitopes showing a significant VaxiJen score (highest score = 1.5485 and lowest score = 0.4551). We found the lowest number of 9-mer CTL antigenic epitopes in B.1.429 (approximately three), with all showing a significant to mild antigen-related VaxiJen score (highest score = 0.4587 and lowest score = 0.6465; Figure 3A).

We performed a comparative assessment and characterization of the 9-mer CTL antigenic epitopes in the S-glycoprotein chain of the Wuhan strain and the B.1.351, B.1.1.28/triple mutant, B.1.1.7, and B.1.429 variants (Table 1). However, during the comparative assessment and characterization, we found that some of the 9-mer CTL antigenic epitopes of the Wuhan strain were also found in particular parts of the S-glycoprotein in the other four variants.

**Table 1:** Identification and comparative analysis of 9 mer CTL epitopes of Wuhan strain and B.1.351, B.1.1.28/triple mutant, B.1.1.7, B.1.429 variant.

Variants name	Epitopes	Epitopes position		VaxiJen score
		S-protein domain	S-protein chain	
B.1.351	FANPVLPFN	NTD	79–87	0.2328 (Non-antigen)
	GLPQGFSAL	NTD	215–223	0.1868 (Non-antigen)
	NIADYNYKL	RBD	417–425	1.5325 (Antigenic)
	KGFNCYFPL	RBD	484–492	0.5711 (Antigenic)
	VENSVAYSN	SD2	701–709	0.4171 (Antigenic)
B.1.1.28/triple mutant	SQCVNLTTR	NTD	13–21	1.5476 (Antigenic)
	SPGSASSVA	SD1	680–688	0.4280 (Antigenic)
	FQPTYGVGY	RBD	497–505	0.4935 (Antigenic)
	NIADYNYKL	RBD	417–425	1.0536 (Antigenic)
B.1.1.7	EILDITPCS	SD1	583–591	0.4935 (Antigenic)
	FQPTYGVGY	RBD	497–505	1.5485 (Antigenic)
	EGFNCYFPL	RBD	481–489	0.5453 (Antigenic)
	RDIDDTTDA	SD1	564–572	0.7902 (Antigenic)
	FTISVTTEI	SD2	715–723	0.8535 (Antigenic)
	IITTHNTFV	CD	1,111–1,119	0.4551 (Antigenic)
B.1.429	CMESEFRVY	NTD	152–160	0.4587 (Antigenic)
	RYRLFRKSN	RBD	452–460	−0.6465 (Non-antigen)
	FQFCNDPFL	NTD	132–140	−0.2493 (Non-antigen)
Wuhan	VVFLHVTYV	HR2	1,060–1,068	1.5122 (Antigenic)
	GKQGNFKNL	NTD	181–189	1.0607 (Antigenic)
	GIYQTSNFR	NTD	311–319	0.5380 (Antigenic)
	VSPTKLNDL	RBD	382–390	1.4610 (Antigenic)
	FKNHTSPDV	CD	1,156–1,164	0.4846 (Antigenic)

**Figure 4:**
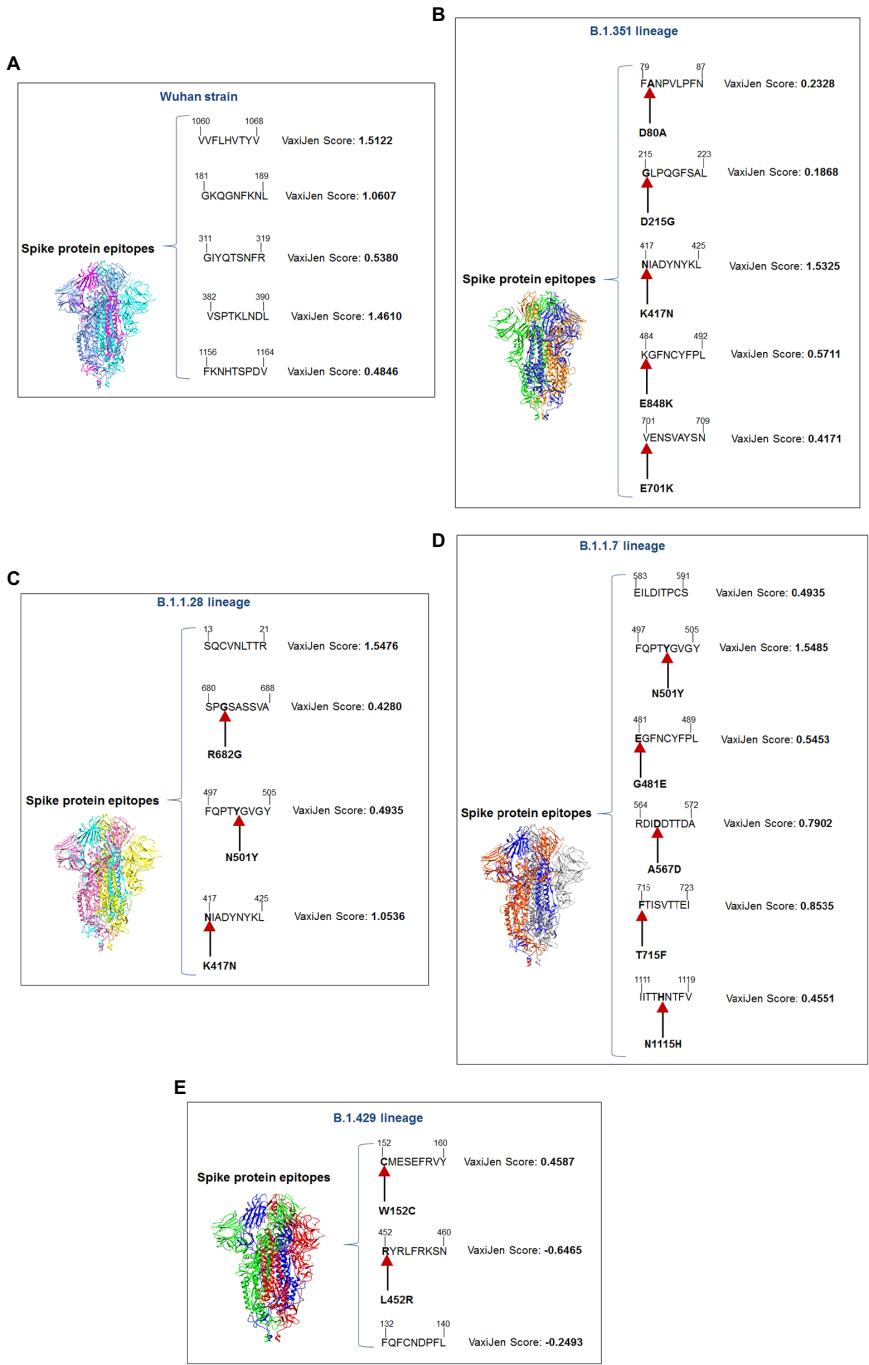
A schematic diagram of significant 9 mer CTL epitopic landscape of S-glycoprotein of Wuhan strain and B.1.351, B.1.1.28/triple mutant, B.1.1.7, and B.1.429 variant. The figure also shows the significant mutations present in the 9 mer CTL epitopic regions. **(A)** A schematic diagram of 9 mer CTL antigenic epitopes identified of S-glycoprotein in Wuhan strain. **(B)** A schematic diagram of 9 mer CTL antigenic epitopes recognized of S-glycoprotein in the B.1.351 variant with significant mutations in the epitopic regions. **(C)** A schematic diagram of 9 mer CTL antigenic epitopes identified of S-glycoprotein in B.1.1.28/triple mutant variant with substantial mutations in the epitopic areas. **(D)** A schematic diagram of 9 mer CTL antigenic epitopes recognized of S-glycoprotein in B.1.1.7 variant with significant mutations in the epitopic regions. **(E)** A schematic diagram of 9 mer CTL antigenic epitopes identified of S-glycoprotein in B.1.429 variant with substantial mutations in the epitopic regions.

Our study observed that the Wuhan strain contained five 9-mer CTL epitopes. One epitope with residue positions 1,060–1,068 showed the highest antigenic score (1.5122; Figure 4A). Similarly, the B.1.351 variant showed five significant mutation 9-mer CTL epitopes. The 9-mer CTL epitope with residue positions 417–425 showed the highest antigenic score (1.5325) and contained the K417N mutation (Figure 4B). This mutation might play a crucial role in providing the highest antigenicity for this particular epitope.

We found four 9-mer CTL epitopes in B.1.1.28/triple mutant. Among the epitopes, the highest antigenic score was 1.5476 in the 9-mer CTL epitope, where no mutations were observed (Figure 4C). The other three epitopes had significant mutations. The second 9-mer CTL epitope (epitopic residue positions 680–688) contained the R682G mutation, and the third epitope (epitopic residue positions 497–505) contained the N501Y mutation. The last one (epitopic residue positions 417–425) had a K417N mutation.

The B.1.1.7 variant contained six 9-mer CTL epitopes. The epitope with residue positions 497–505 showed the highest antigenicity (antigenic score 1.5485). This CTL epitope contained the N501Y mutation (Figure 4D), which might substantially provide the antigenicity for this CTL epitope. Other epitopes included different mutations [G481E (epitopic residue positions 481–489), A567D (epitopic residue positions 564–572), T715F (epitopic residue positions 715–723), and N1115H (epitopic residue positions 1,111–1,119)].

The 9-mer CTL epitopes analysis found that the B.1.429 variant contained one significant antigenic CTL epitope (antigenic score 0.4587). This epitope, with residue positions 152–160, consisted of a substantial mutation (W152C). In this variant, we also found two non-antigenic epitopes: the first one with residue positions 452–460 (antigenic score−0.6465) and the second one with residue positions 132–149 (antigenic score−0.2493; Figure 4E). The CTL epitope with residue positions 452–460 contained the L452R mutation, which might help generate a negative score for the epitope. Therefore, in this variant, antigenicity loss was noted in the 9-mer CTL epitopes.

### Comparative mapping of significant 9-mer CTL epitope of the S-glycoprotein of the Wuhan strain and the B.1.351, B.1.1.28/triple mutant, B.1.1.7, and B.1.429 variants

**Figure 5:**
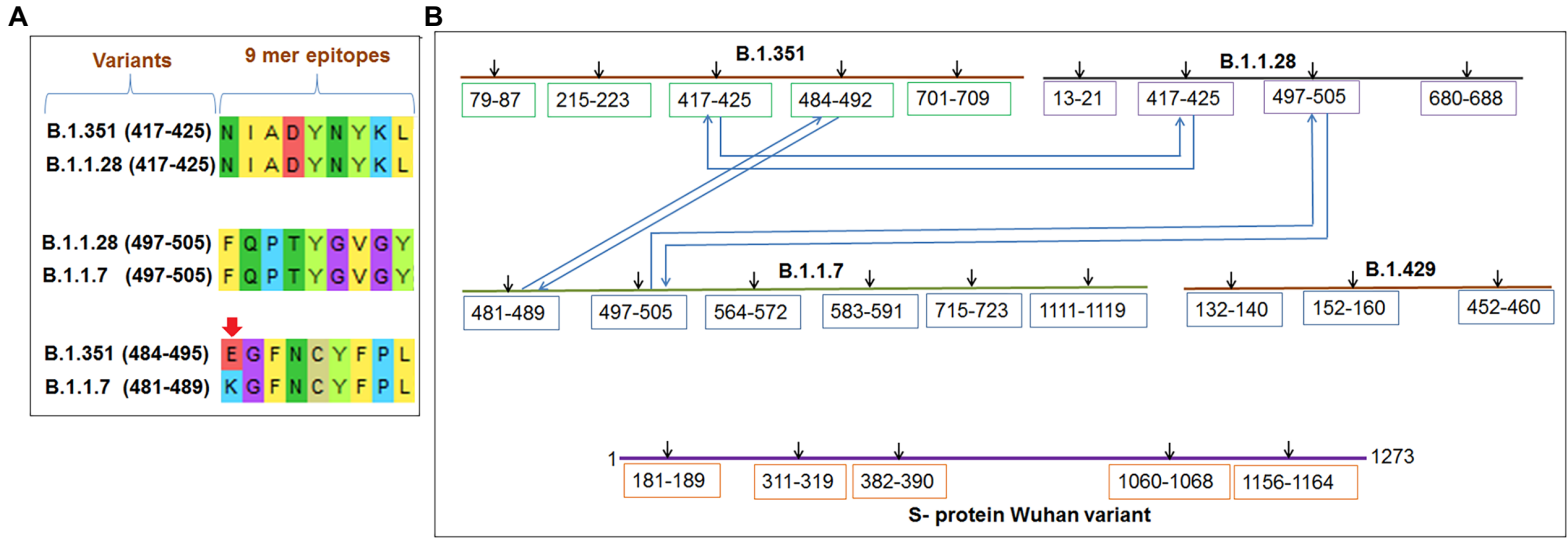
Comparative mapping of significant 9 mer CTL epitope of S-glycoprotein of Wuhan strain and B.1.351, B.1.1.28/triple mutant, B.1.1.7, and B.1.429 variant. **(A)** Epitopes homology of Wuhan strain and B.1.351, B.1.1.28/triple mutant, B.1.1.7, and B.1.429 variant. **(B)** A schematic diagram shows the similarity between the epitopes among B.1.351, B.1.1.28/triple mutant, B.1.1.7, and B.1.429 variant.

Comparative sequence homology analysis of the 9-mer CTL epitopes using S-glycoprotein from the Wuhan strain and B.1.351, B.1.1.28/triple mutant, B.1.1.7, and B.1.429 variants showed some epitopic homology among significant VOCs/VOI/others. Sequence homology was observed between the epitopic regions of the S-glycoprotein in the two variants such as B.1.351 and B.1.1.7. We found epitopic homology between the 481–489 residues in epitopic part of the B.1.1.7 variant and the 484–492 residues in epitopic part of the B.1.351 variant. Similarly, we also found high sequence homology between the 497–505 epitopic part of the B.1.1.7 variant and 497–505 residues in epitopic part of the B.1.1.28/triple mutant variant. A substantial epitopic homology was observed between the 417–525 epitopic region of the B.1.351 variant and the 417–525 epitopic part of the B.1.1.28/triple mutant variant (Figure 5A). The evolutionary process is attributed to creating the epitopic homology of variants, which determines their antigenicity. However, epitopic homology was not observed in the Wuhan strain and B.1.429 variant (Figure 5B).

### MSA using the identified CTL epitopes (9-mer, 15-mer, and 20-mer) of S-glycoprotein of the Wuhan strain and B.1.351, B.1.1.28/triple mutant, B.1.1.7, and B.1.429 variants

**Figure 6:**
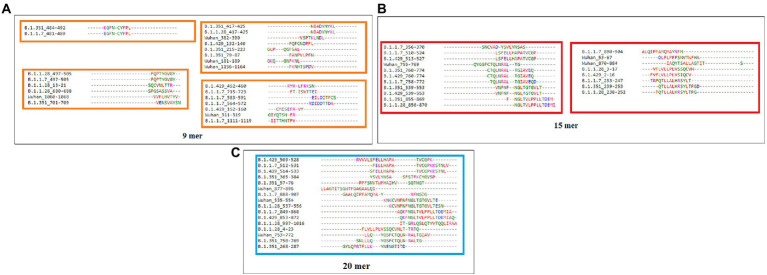
Epitope alignment of 9 mer, 15 mer, 20 mer CTL antigenic epitopes present in the S-glycoprotein of Wuhan strain and B.1.351, B.1.1.28/triple mutant, B.1.1.7, and B.1.429 variant. **(A)** Epitope alignment shows four significant blocks from 9 mer CTL antigenic epitopes. **(B)** Epitope alignment shows two significant blocks from 15 mer CTL antigenic epitopes. **(C)** Epitope alignment shows one significant block from 20 mer CTL antigenic epitopes.

Sequence alignment of 9-mer, 15-mer, and 20-mer CTL epitopes of the S-glycoprotein of the Wuhan strain and B.1.351, B.1.1.28/triple mutant, B.1.1.7, and B.1.429 variants were examined. The 9-mer CTL epitopes showed four blocks (Figure 6A), whereas the 15-mer CTL epitopes showed two blocks (Figure 6B) and the 20-mer CTL epitopes showed one block (Figure 6C).

### Cluster analysis of 9-mer CTL epitopes of S-glycoprotein of the Wuhan strain and B.1.351, B.1.1.28/triple mutant, B.1.1.7, and B.1.429 variants

**Figure 7:**
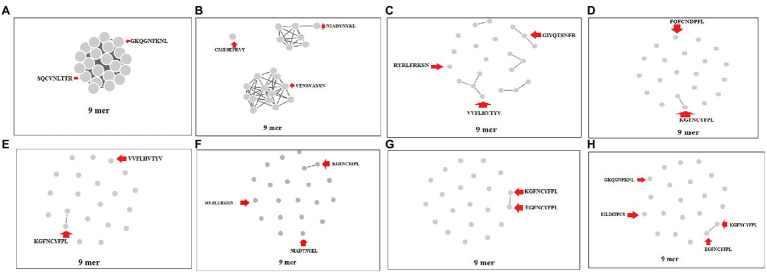
Cluster formation of 9 mer CTL epitopes of S-glycoprotein of Wuhan strain and B.1.351, B.1.1.28/triple mutant, B.1.1.7, and B.1.429 variant using the threshold 10% level to 80% level. **(A)** Cluster formation at 10% level. **(B)** Cluster formation at 20% level. **(C)** Cluster formation at 30% level. **(D)** Cluster formation at 40% level. **(E)** Cluster formation at 50% level. **(F)** Cluster formation at 60% level. **(G)** Cluster formation at 70% level. **(H)** Cluster formation at 80% level.

We performed a cluster analysis of the 9-mer CTL epitopes of the S-glycoprotein of the Wuhan strain and B.1.351, B.1.1.28/triple mutant, B.1.1.7, and B.1.429 variants at different threshold levels (10 to 80% level). We used 23 of the 9-mer CTL epitopes for cluster formation. We found that one dense cluster was formed at the 10% level (Figure 7A), which had a circular shape, and approximately 23 nodes were visible.

We found that three clusters formed at the 20% level, containing one, seven, and 13 nodes (Figure 7B). We observed five prominent cluster formations at the 30% level, which had two, three, three, three, and four nodes (Figure 7C). We found one significant cluster at the 40% level that contained two nodes (Figure 7D), with the other nodes individually scattered. We found one crucial cluster creation at the 50% level, including two nodes (Figure 7E), and the other nodes were individually scattered. We observed one significant cluster at the 60% level, containing two nodes, with the other nodes individually scattered (Figure 7F). We evaluated the cluster formation at the 70% level, which showed one significant cluster, containing two nodes and several nodes individually scattered (Figure 7G). Finally, we evaluated the cluster formation at the 80% level, which showed one significant cluster containing two nodes and several nodes positioned in a scattered manner (Figure 7H).

### Cluster analysis of 15-mer CTL epitopes of the Wuhan strain and B.1.351, B.1.1.28/triple mutant, B.1.1.7, and B.1.429 variants

We performed a cluster analysis of the 15-mer CTL epitopes of the S-glycoprotein of the Wuhan strain and B.1.351, B.1.1.28/triple mutant, B.1.1.7, and B.1.429 variants at different threshold levels (10 to 80% level). We examined 19 numbers of the 15-mer CTL epitopes for cluster formation. We found a dense cluster formation at the 10% level, with a circular shape, and approximately 16 nodes were visible (Supplementary Figure S1A).

We found one significant cluster at the 20% level, containing 15 nodes, and observed another single cluster with one node (Supplementary Figure S1B). We found three prominent clusters at the 30% level, containing three, three, and four nodes (Supplementary Figure S1C). We found four significant clusters at the 40% level, having three, three, three, and two, and the other nodes were individually scattered (Supplementary Figure S1D). At the 50% level, we identified four critical clusters containing three, three, two, and two nodes and several nodes individually scattered (Supplementary Figure S1E). We observed four major clusters at the 60% level, containing two, two, three, and three nodes and several other nodes were independently scattered (Supplementary Figure S1F). We evaluated the cluster formation at the 70% level, which showed four significant clusters containing three, two, two, and two nodes, and several nodes individually scattered (Supplementary Figure S1G). Finally, we evaluated the cluster formation at the 80% level, which showed four significant clusters, containing two, two, two, and three nodes, and numerous nodes separately situated in a scattered manner (Supplementary Figure S1H).

### Cluster analysis of the 20-mer CTL epitopes of S-glycoprotein of the Wuhan strain and B.1.351, B.1.1.28/triple mutant, B.1.1.7, and B.1.429 variants

We performed cluster analysis of the 20-mer CTL epitopes of S-glycoprotein of the Wuhan strain and B.1.351, B.1.1.28/triple mutant, B.1.1.7, and B.1.429 variants at different threshold levels (minimum sequence identity threshold 10 to 80% level). We examined 16 numbers of the 20-mer CTL epitopes for cluster formation. We found one dense cluster formation at the 10% level, which had a circular shape, and approximately 16 nodes were visible (Supplementary Figure S2A). All 20-mer CTL epitopes participated in the cluster formation.

We found two significant clusters at the 20% level, containing four and 12 nodes (Supplementary Figure S2B). We found four prominent clusters at the 30% level, including four, two, two, and three nodes, and the other nodes were individually scattered (Supplementary Figure S2C).

We found four significant clusters that were generated at the 40% level, containing two, two, two, and three nodes and some other nodes were individually scattered (Supplementary Figure S2D). We observed four significant clusters created at the 50% level, containing three, two, two, and two nodes and several nodes positioned individually in a scattered manner (Supplementary Figure S2E). We observed four major clusters at the 60% level, containing two, two, two, and three nodes and some other nodes were located independently in a scattered manner (Supplementary Figure S2F). We evaluated the cluster formation at the 70% level, which showed four significant clusters, containing two, two, two, and three nodes and several nodes were individually scattered (Supplementary Figure S2G). We evaluated the cluster formation at the 80% level, which showed four significant clusters, containing two, two, two, and two nodes and numerous single nodes were situated in a scattered manner (Supplementary Figure S2H).

### Analysis of the CD spectra of S-glycoprotein of the Wuhan strain and B.1.351, B.1.1.28/triple mutant, B.1.1.7, and B.1.429 variants

**Figure 8:**
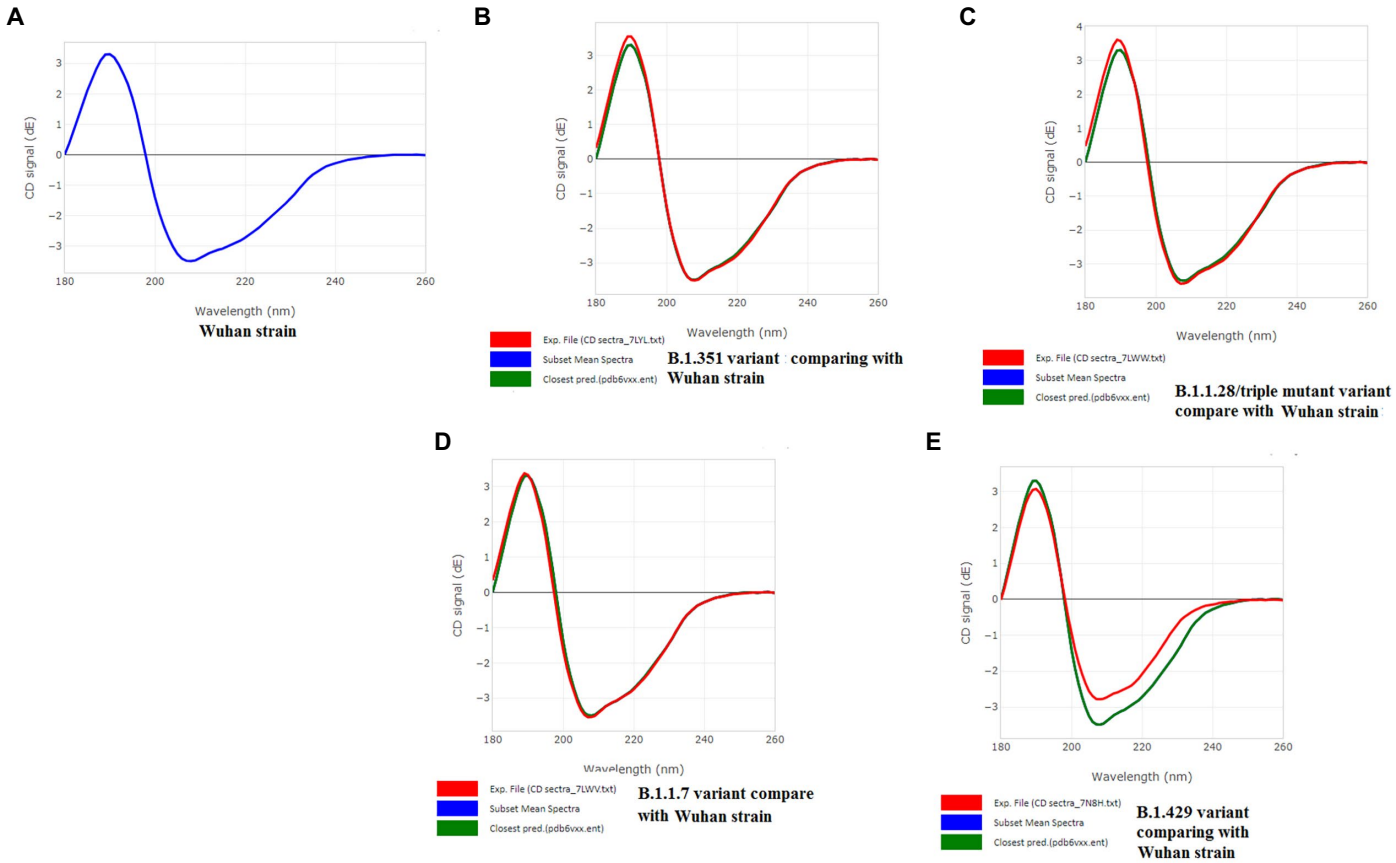
CD spectra of S-glycoprotein of the Wuhan variant and B.1.351, B.1.1.28/triple mutant, B.1.1.7, B.1.429 variants. **(A)** CD spectra of S-glycoprotein of the Wuhan strain **(B)** Resemblance of CD spectra of S-glycoprotein of the Wuhan strain and B.1.351. **(C)** The resemblance of CD spectra of S-glycoprotein of the Wuhan strain and B.1.1.28/triple mutant. **(D)** The resemblance of CD spectra of S-glycoprotein of the Wuhan strain and B.1.1.7. **(E)** The resemblance of CD spectra of S-glycoprotein of the Wuhan strain and B.1.429.

**Figure 9:**
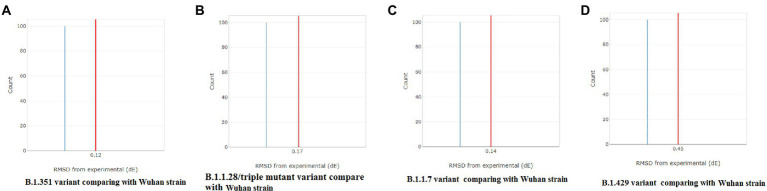
Comparison of RMSD between the CD spectra of the S-glycoprotein Wuhan variant and other variants. **(A)** The resemblance of RMSD between the CD spectra of S-glycoprotein Wuhan strain and B.1.351. **(B)** The resemblance of RMSD between the CD spectra of S-glycoprotein Wuhan strain and B.1.1.28/triple mutant. **(C)** The resemblance of RMSD between the CD spectra of the S-glycoprotein Wuhan strain and B.1.1.7. **(D)** The resemblance of RMSD between the CD spectra of S-glycoprotein Wuhan strain and B.1.429.

A CD spectrum is essential for analyzing the proper secondary structure and fold detection of a protein (Micsonai et al., 2015). We generated the computer-simulated CD spectra of S-glycoprotein of the Wuhan strain and B.1.351, B.1.1.28/triple mutant, B.1.1.7, and B.1.429 variants. The spectrum of the viral glycoprotein of the Wuhan strain showed both positive and negative bands. The CD spectra showed a positive band at approximately 198 nm, comprising a maximum positive peak at about 190 nm, and showed a negative band from 198 nm to 225 nm with a maximum negative peak at 207–210 nm (Figure 8A). We analyzed the CD spectra of the B.1.351 variant, where we used the Wuhan strain as a control or the closest CD spectrum to the experimental protein (green spectra). Here, we used the S-glycoprotein of the B.1.351 variant as the experimental protein, and the generated spectrum is shown in red (Figure 8B). Both CD spectra showed the same pattern, with the maximum positive peak greater for the B.1.351 variant than for the Wuhan strain. The study evaluated the root-mean-square deviation (RMSD) variation of the control or closest CD spectrum and experimental CD spectrum (B.1.351 variant), which was 0.12 (Figure 9A; Supplementary Table S3). We analyzed the CD spectrum of the B.1.1.28/triple mutant variant using the same methodology as described above (Figure 8C). Both CD spectra (Wuhan strain and B.1.1.28/triple mutant variant) showed a similar pattern. The maximum positive peak was slightly more remarkable in the B.1.1.28/triple mutant variant than in the Wuhan strain, with an RMSD variation of 0.17 (Figure 9B). We generated the CD spectrum of the B.1.1.7 variant and compared it to the Wuhan strain (Figure 8D), and both CD spectra showed identical patterns. Maximum positive and maximum negative peaks were the same. The RMSD variation was 0.14 (Figure 9C). Finally, we generated the CD spectrum of the B.1.429 variant and compared it to the Wuhan strain (Figure 8E), with both CD spectra showing identical patterns. A lower negative peak was observed for the CD signal of the B.1.429 variant than the Wuhan strain, and the RMSD variation was 0.45 (Figure 9D). We also analyzed the secondary structure pattern of the Wuhan strain, B.1.351, B.1.1.28/triple mutant, B.1.1.7, and B.1.429 variants to calculate the beta-sheet (β-sheet) percentages, which were 26.21% (Supplementary Figure S3A), 24.84% (Supplementary Figure S3B), 24.84% (Supplementary Figure S3C), 24.46% (Supplementary Figure S3D), and 32.19%, respectively (Supplementary Figure S3E; Supplementary Table S4). Therefore, all proteins were β-sheet-rich. However, more β-sheets were found in B.1.429 than in the Wuhan strain and the B.1.351, B.1.1.28/triple mutant, and B.1.1.7 variants (Supplementary Figure S4A). In addition, more β turns (9.71%) were found in B.1.429 than in the Wuhan strain and the B.1.351, B.1.1.28/triple mutant, and B.1.1.7 variants (Supplementary Figure S4B).

The CD spectra of proteins were applied to evaluate proteins’ secondary structure pattern, binding properties, and folding shape. It is one of the rapid methods for assessing proteins’ secondary structure pattern, binding properties, and folding shape.

Parsons et al. (2019) have evaluated the RBD protein secondary structure of avian coronavirus by CD spectra analysis (Parsons et al., 2019). In this analysis, the spectral bandwidth has shown diverse values regarding nanometer scale, specific temperature, etc., of RBD proteins of glycosylation-site and non-glycosylation-site variants.

In our study, we applied the PDBMD2CD server to generate the *in silico* CD spectra (wavelength of the CD signal) using PDB files of S-glycoprotein of the Wuhan strain and significant variants.

The secondary structure pattern of S-glycoprotein of studied SARS-CoV-2 (Wuhan strain and other variants) features are stabilized by the amino acid residues turn tendencies as well as the cross-strand interactions among the sequences flanking the beta-turns. Researchers also showed that the specific β-turns of protein have a crucial role in interacting with the T-cell receptor (TCR), leading the MHCII-peptide-TCR complex to induce a suitable immune response (Bermudez et al., 2018). Subsequently, the beta-sheets also significantly impact on the managing the affinity of MHC class II binding sites in superantigens (Al-Shangiti et al., 2004). However, in the case of SARS-CoV-2 S-glycoprotein, no such work has been reported. But, our bioinformatics tools have shown the proper interactions of epitopic peptides (9-mer) from S-glycoprotein with TCR. These tools have established the vital role of beta-sheets and beta-turns in forming the SAg-like region.

### 3D model generation of the 9-mer CTL epitopic regions

**Figure 10:**
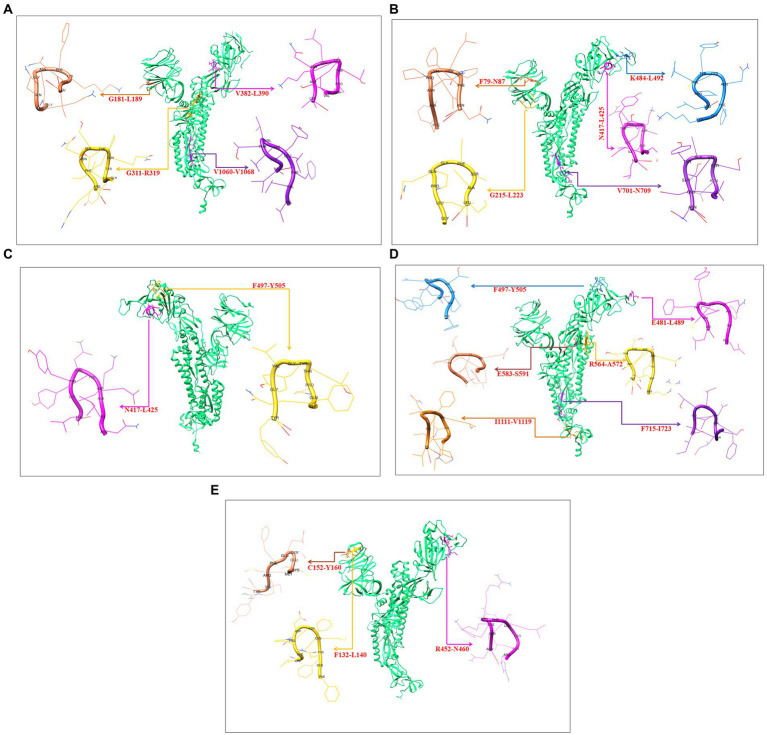
Generated three-dimensional (3D) model of 9 mer CTL epitopic regions of the Wuhan strain and B.1.351, B.1.1.28/triple mutant, B.1.1.7, B.1.429 variants and their position in 3D model of S-glycoprotein. **(A)** 3D model of 9 mer CTL epitopic regions of the Wuhan strain and their position in 3D model of S-glycoprotein. **(B)** 3D model of 9 mer CTL epitopic regions of the B.1.351 variant and their position in 3D model of S-glycoprotein. **(C)** 3D model of 9 mer CTL epitopic regions of the B.1.1.28/triple mutant variant and their position in 3D model of S-glycoprotein. **(D)** 3D model of 9 mer CTL epitopic regions of the B.1.1.7 variant and their position in 3D model of S-glycoprotein. **(E)** 3D model of 9 mer CTL epitopic regions of the B.1.429 variant and their position in the 3D model of S-glycoprotein.

3D models were created for all 9-mer CTL epitopes, and their epitopic regions were located in the S-glycoprotein. We generated four 3D models for the 9-mer CTL epitopes of the Wuhan strain: G181-L189, G311-R319, V382-L390, and V1060-1068 (Figure 10A); four 3D models for the B.1.351 variant: F79-N87, G215-L223, N417-L425, K484-L492, and V701-N709 (Figure 10B); two 3D models for the B.1.1.28/triple mutant variants: N417-L425 and F497-Y505 (Figure 10C); six 3D models for the B.1.1.7 variant: E481-L489, F497-Y505, R564-A572, E583-S591, F715-I723, and I1111-V1119 (Figure 10D); and three 3D models for the B.1.429 variant: F132-L140, C152-Y160, and R452-N460 (Figure 10E).

### Mimic phage-displayed peptides for 9-mer CTL epitopes of SARS-CoV-2 S-glycoprotein

**Figure 11:**
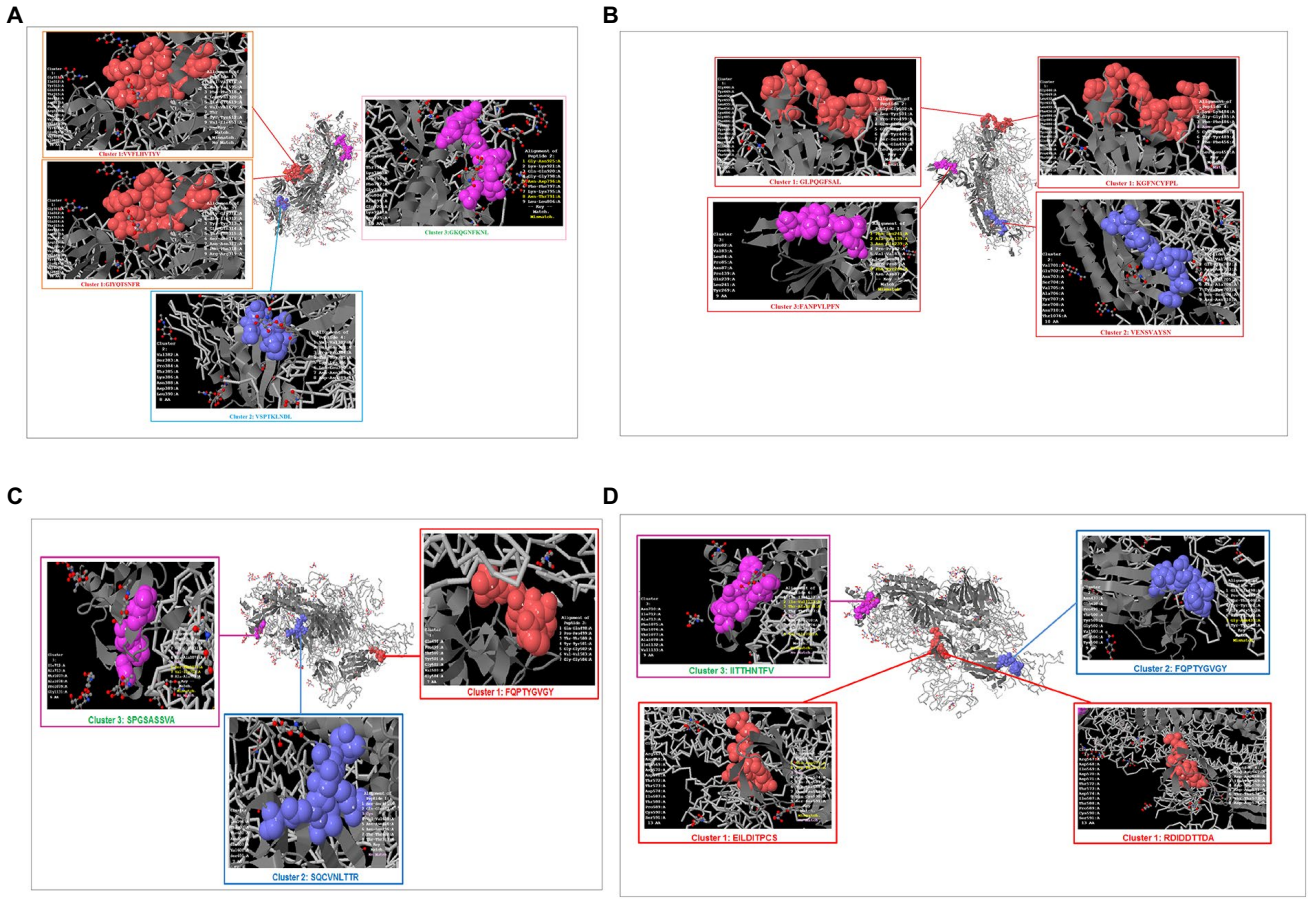
Phage-displayed peptides that mimic S-glycoprotein of SARS-CoV-2 9 mer CTL epitopes of the Wuhan strain and B.1.351, B.1.1.28/triple mutant, B.1.1.7, B.1.429 variants. **(A)** Phage-displayed peptides that mimic S-glycoprotein of 9 mer CTL epitopes of the Wuhan strain and the cluster epitopes. **(B)** Phage-displayed peptides that mimic S-glycoprotein of 9 mer CTL epitopes of the B.1.351 variant and the cluster epitopes. **(C)** Phage-displayed peptides that mimic S-glycoprotein of 9 mer CTL epitopes of the B.1.1.28/triple mutant variant and the cluster epitopes. **(D)** Phage-displayed peptides that mimic S-glycoprotein of 9 mer CTL epitopes of B.1.1.7 and the cluster epitopes.

Previous studies have identified unique phage-displayed peptides that mimic different pathogen epitopes, such as *Mycobacterium tuberculosis* (Wang et al., 2016) and the hepatitis E virus (Larralde and Petrik, 2017). The present study aimed to determine the mimic 9-mer CTL epitopes from the S-glycoprotein of SARS-CoV-2 from phage-displayed peptides. A random peptide library was scanned, and the selected peptides were supposed to mimic the 9-mer CTL epitopes of S-glycoprotein of this virus, which showed a list of clusters in the Wuhan, B.1.351, B.1.1.28/triple mutant, and B.1.1.7 variants. The Wuhan strain showed three mimic clusters with four different 9-mer epitopes (Figure 11A). The first mimic cluster was formed with two 9-mer epitopes (VVFLHVTYV and GIYQTSNFR), the second mimic cluster was formed with one 9-mer epitope (VSPTKLNDL), and the third mimic cluster was formed with one 9-mer epitope (GKQGNFKNL).

The B.1.351 variant showed three mimic clusters with four 9-mer epitopes (Figure 11B). The first mimic cluster consisted of two 9-mer epitopes (GLPQGFSAL and KGFNCYFP), the second was formed with one 9-mer epitope (VENSVAYSN), and the third included one 9-mer epitope (FANPVLPFN).

The B.1.1.28/triple mutant variant showed three mimic clusters with three different 9-mer epitopes (Figure 11C). The first mimic cluster consisted of one 9-mer epitope (FQPTYGVGY), the second was formed with one 9-mer epitope (SQCVNLTTR), and the third was formed with one 9-mer epitope (SPGSASSVA).

Finally, the B.1.1.7 variant showed three mimic clusters with four 9-mer epitopes (Figure 11D). The first mimic cluster had two 9-mer epitopes (EILDITPCS and RDIDDTTDA), the second was formed with one 9-mer epitope (FQPTYGVGY), and the third consisted of one 9-mer epitope (IITTHNTFV).

### SAg-like region from S-glycoprotein of SARS-CoV-2 of the Wuhan strain and B.1.351, B.1.1.28/triple mutant, B.1.1.7, and B.1.429 variants

**Figure 12:**
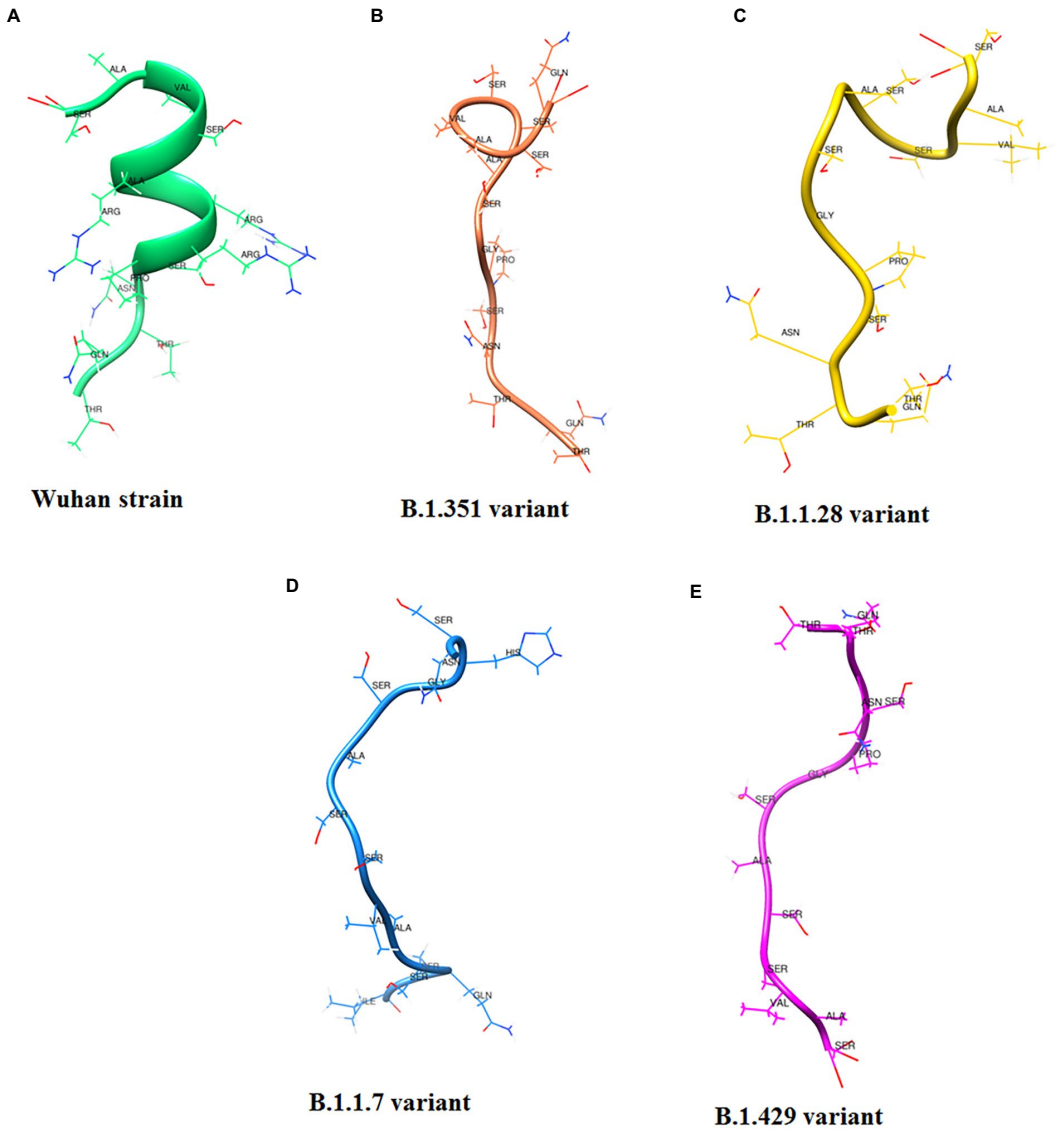
3D model of SAg-like region from S-glycoprotein of Wuhan strain and B.1.351, B.1.1.28/triple mutant, B.1.1.7 and B.1.429 variants. **(A)** 3D model of SAg-like region from S-glycoprotein of Wuhan strain. **(B)** 3D model of SAg-like region from S-glycoprotein of the B.1.351 variant which was generated same SAg-like region from S-glycoprotein of Wuhan strain. **(C)** 3D model of SAg-like region from S-glycoprotein of the B.1.1.28/triple mutant variant which was generated same SAg-like region from S-glycoprotein of Wuhan strain. **(D)** 3D model of SAg-like region from S-glycoprotein of the B.1.1.7 variant which was generated same SAg-like region from S-glycoprotein of Wuhan strain. **(E)** 3D model of SAg-like region from S-glycoprotein of the B.1.429 variant which was generated same SAg-like region from S-glycoprotein of Wuhan strain.

Following Cheng et al. (2020) study, we developed a 3D structural model of the SAg-like part of the S-glycoprotein of SARS-CoV-2 of the Wuhan strain (Figure 12A; Baú et al., 2006; Cheng et al., 2020). We also developed a 3D structure model using the same region (SAg-like region) from the S-glycoprotein of this virus of the different variants, i.e., B.1.351 (Figure 12B), B.1.1.28/triple mutant (Figure 12C), B.1.1.7 (Figure 12D), and B.1.429 (Figure 12E).

### Interaction between SAg-like region of the viral glycoprotein and T cell receptor (TCR)

**Figure 13:**
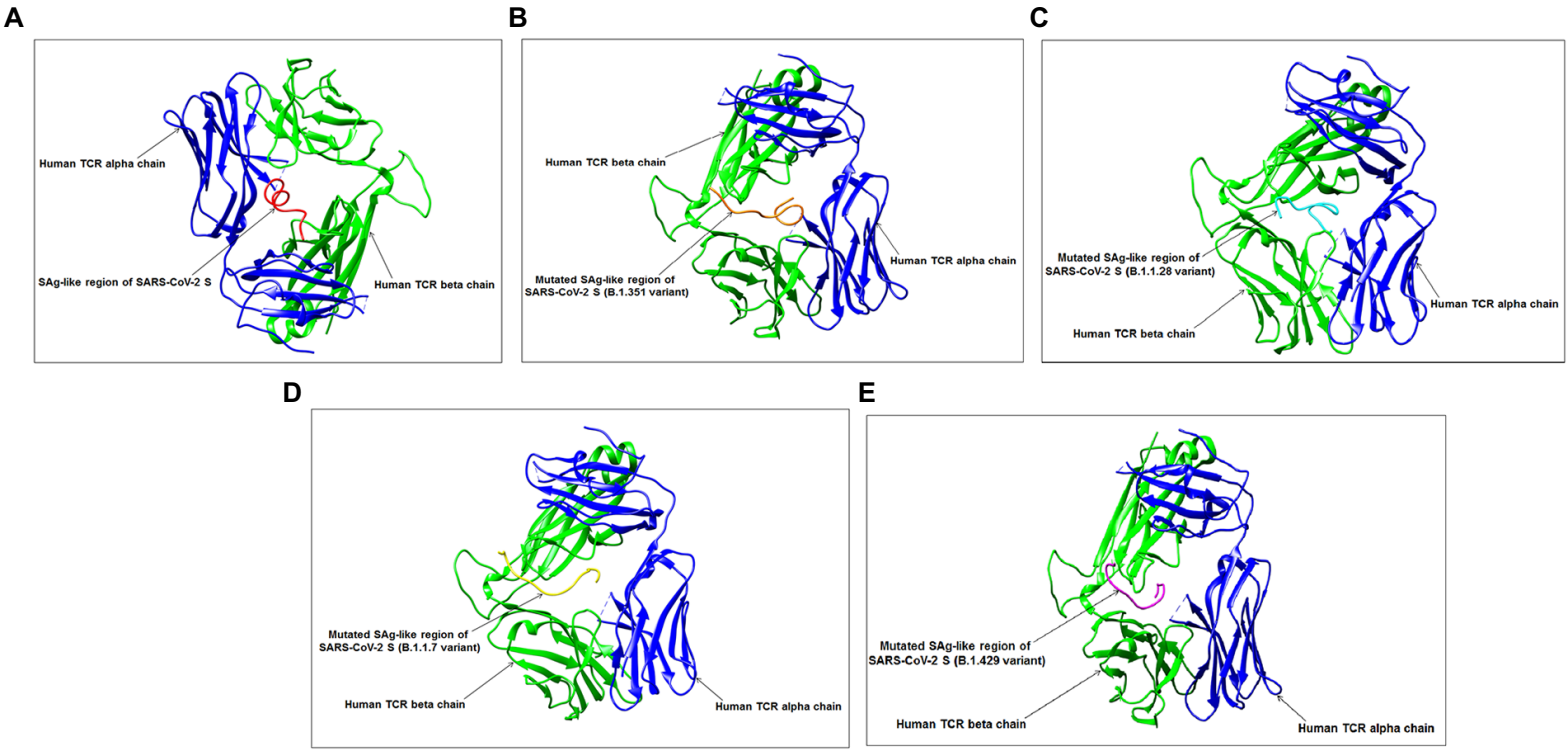
Interaction between SAg-like region from S-glycoprotein of Wuhan strain, B.1.351, B.1.1.28/triple mutant, B.1.1.7, B.1.429 variants and TCR. **(A)** Interaction between SAg-like region of Wuhan strain and TCR. **(B)** Interaction between SAg-like part of B.1.351 variant and TCR. **(C)** Interaction between SAg-like part of the B.1.1.28/triple mutant variant and TCR. **(D)** Interaction between the SAg-like part of the B.1.1.7 variant and TCR. **(E)** Interaction between the SAg-like part of the B.1.429 variant and TCR.

The present study illustrated the interaction with our developed 3D structural model of the SAg-like region of the S-glycoprotein of the Wuhan strain and B.1.351, B.1.1.28/triple mutant, B.1.1.7, and B.1.429 variants with human TCR. These interactions can be seen in Figures 13A–E and showed that the SAg-like region interacted with the beta chain of the human TCR.

### Identification of the partial SAg-like part of the viral S-glycoprotein of the Wuhan strain and B.1.351, B.1.1.28/triple mutant, B.1.1.7, and B.1.429 variants

**Figure 14:**
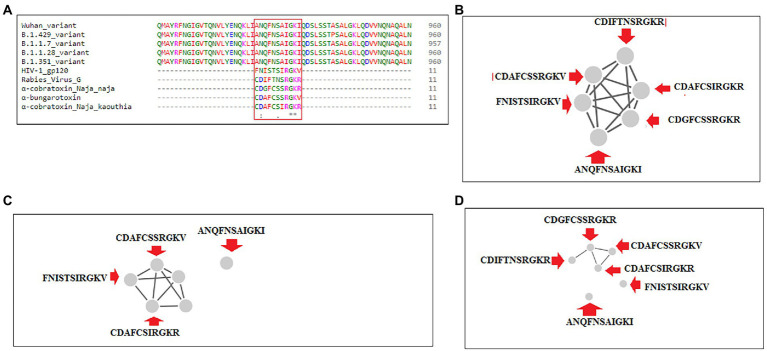
Another partial SAg-like region from S-glycoprotein of SARS-CoV-2 of Wuhan strain, B.1.351, B.1.1.28/triple mutant, B.1.1.7, B.1.429 variants shows the sequence similarity with an α-cobra toxin (*Naja naja*),α-bungarotoxin, Rabies Virus G Protein (189–199), α-cobra toxin (*Naja kaouthia*), and HIV-1 gp120 (164–174) and their cluster formation. **(A)** Sequence similarity of superantigens (α-cobra toxin (*Naja naja*),α-bungarotoxin, Rabies Virus G Protein (189–199), α-cobra toxin (*N. kaouthia*), and HIV-1 gp120 (164–174)), Wuhan variant, and significant VOCs/VOI (B.1.351, B.1.1.28/triple mutant, B.1.1.7, B.1.429) shows a partial super SAg-like region. **(B)** Cluster (with these MSA sequences) at 30% level. **(C)** Cluster (with these MSA sequences) at 40% level. **(D)** Cluster (with these MSA sequences) at 70% level.

Sequence alignment was performed using significant SAgs [α-cobra toxin (*N. naja*), α-bungarotoxin, rabies virus G protein (189–199), and α-cobra toxin (*N. kaouthia*)], and HIV-1 gp120 (164–174), Wuhan strain and B.1.351, B.1.1.28/triple mutant, B.1.1.7, and B.1.429 variants. We observed a partial SAg-like part (ANQFNSAIGKI) of the S-glycoprotein of this virus in the Wuhan strain and B.1.351, B.1.1.28/triple mutant, B.1.1.7, and B.1.429 variants, as depicted in Figure 14A.

We developed a cluster with these MSA sequences at the 30, 40, and 70% levels. We found one cluster with all sequences at the 30% level (Figure 14B). However, the SAg-like region was detached from the central cluster at 40% (Figure 14C) and 70% levels (Figure 14D).

## Discussion

MHC-I-restricted CTLs play a significant role in controlling viral infections. CD8^+^ T cells perform a crucial role in the clearance of infections of this virus (acute model) in the lungs (Schmidt and Varga, 2018) and are activated and differentiated in patients with severe COVID-19 (Chen and Wherry, 2020). Bange et al. (2021) concluded that CD8^+^ T cells might help with the survival of patients with COVID-19 (Bange et al., 2021). Therefore, identifying CTL epitopes is essential for understanding the T cell activation mechanism and assisting epitope-driven vaccine design.

The identification and characterization of the CTL 9-mer epitope have a substantial importance in medical immunology. The identification of peptide epitopes can be helpful in the production of vaccines against microorganisms that have very little growth in the medium, as well as microorganisms whose antigenic regions are not precisely detected during the typical immune system infection (Andreatta and Nielsen, 2018; Rencilin et al., 2021). Therefore, finding considerable numbers of CTL epitopes is a necessary process for vaccine design, and those CTL epitopes consist of the binding motifs of the known class I MHC. Still, even the epitopes do not conform precisely to consensus motifs or vary in length. To do this with conventional peptide synthesizers is costly and impractical because of the large number of peptides, and a considerable amount of testing is required. Therefore, these advanced strategies for epitope mapping offer a more precise solution than the initial CTL epitope screening experiments rather than other aspects (Soria-Guerra et al., 2015).

In the present study, CTL epitopic regions of S protein were analyzed using antigenic scores. We assessed the antigenicity of the wild type (Wuhan strain) and mutant S-glycoprotein of four variants (B.1.351, B.1.1.28/triple mutant, B.1.1.7, and B.1.429). The analysis of 9-mer CTL epitope regions of S-glycoprotein showed that the B.1.1.7 variant had an elevated antigenicity compared to other variants and the Wuhan strain for the 9-mer CTL epitopes and average CTL epitopes (average number of three epitope types). The RBD region also showed the highest numbers of 9-mer CTL epitope and 15-mer CTL epitope and an average number of CTL epitopes for the B.1.1.7 variant.

CTL 9-mer epitope sequences in the viral protein can be identified using the IEDB server. This is a standard method (Islam et al., 2020). The selected peptide size (9 amino acids) has a potential binding capacity to MHC-I rather than the 15-mer and 20-mer epitopes because these are optimal stimulators for CD8 response and can interact with several HLA-A alleles, which has a considerable binding affinity (Li Pira et al., 2010; Shi et al., 2015). Therefore, our study performed the identification and characterization of the CTL 9-mer epitope of the Wuhan strain and four mutative variants, which has immense importance.

We have used the IEDB epitope cluster analysis tool (Dhanda et., 2018) for the cluster analysis of CTL epitopes (9-mer, 15-mer, and 20-mer) of S-glycoprotein of the Wuhan strain and the B.1.351, B.1.1.28/triple mutant, B.1.1.7, and B.1.429 variants. This tool groups epitopes into clusters based on sequence identity. A cluster is a group of sequences with a sequence similarity more incredible than the minimum sequence identity threshold specified. Our manuscript represents the CTL epitopic peptides as circles, and the solid line connecting two peptides is identified above the specified threshold. All the connected peptides are in a cluster. Here, all the peptides homologous to a certain pre-specified level are clustered together, for example, at the 20% level. In this case, any member of the cluster will be at least 20% homologous to at least one member. However, the approach’s shortcoming is that cluster members might and often are related by levels of homology much lower than 20%. As a result, the cluster provides a clear consensus sequence. Conversely, the singletons are isolated peptides, and those do not share any sequence identity with any other peptides in the given data. However, using this approach, in our work, we have analyzed and offered from a 10% threshold level of sequence identity to the 80% level of CTL epitopes (9-mer, 15-mer, and 20-mer) of mentioned SARS-CoV-2 Wuhan strain and other significant variants.

During cluster formation of 9-mer CTL epitopes, we found one significant cluster formation at the 70% level with two nodes (KGFNCYFPL and EGFNCYFPL), and the other nodes were scattered. Consequently, they did not form a dense cluster at the 70% level and showed a high level of diversity. We found four significant clusters in the 15-mer CTL epitopes and four multiple clusters formed in the 20-mer CTL epitopes at the 70% level. Considering the cluster formation of the 9-mer, 15-mer, and 20-mer CTL epitopes, the 9-mer CTL epitopes showed uniqueness in their cluster formation pattern. These epitopes, which have formed the clusters in higher percentage levels (such as 70% or 80%), might have a similar kind of nature regarding antigenicity and their antigen processing. However, we need to confirm the hypothesis through further experiments. Therefore, understanding the method of cluster formation is of enormous medical significance. Recently, Meckiff et al. (2020) used the cluster formation technique and the epitope mega pool of peptide design for this virus during the COVID-19 infection (Meckiff et al., 2020).

The CD spectra of the S-glycoprotein of the Wuhan strain and B.1.351, B.1.1.28/triple mutant, and B.1.1.7 variants showed almost the same band pattern. On the other hand, we have found a slight difference in the CD spectra of S-glycoprotein of the B.1.429 variant compared to the Wuhan strain. This difference occurred because of the spectral contrast of the B.1.429 variant and Wuhan strain. After comparing the closest and experimental CD spectra, the RMSD variation was calculated. The Wuhan strain and the B.1.351, B.1.1.28/triple mutant, and B.1.1.7 variants had minor differences (0.12–0.17 range). However, the RMSD variation was 0.45 for the B.1.429 variant. A higher RMSD variation occurred because of more spectral differences than the control. This result is in corroboration with the results of Li and Hirst (Li and Hirst, 2020), who attempted to develop a model of SARS-CoV-2 proteins using CD spectra analysis.

The present study found three phage-displayed peptides that mimicked the 9-mer CTL epitopes from the S-glycoprotein of SARS-CoV-2 (Wuhan strain, and B.1.351, B.1.1.28/triple mutant, and B.1.1.7 variants). Previous studies have identified mimic phage-displayed peptides similar to CTL epitopes of SARS-CoV-2, which have immense importance for epitope-based immunotherapy, diagnostics, and vaccine development (Bazan et al., 2012; Wu et al., 2016). Recently, Guo et al. (2021) characterized different B cell epitopes from this virus using a peptide library (phage-displayed; Guo et al., 2021).

Finally, we attempted to understand the interaction pattern of the SAg-like region within the Wuhan strain and TCR, following Cheng et al. (Cheng et al., 2020). An identical portion of the SAg-like part of the Wuhan strain was searched from the B.1.351, B.1.1.28/triple mutant, B.1.1.7, and B.1.429 variants. The hyperinflammatory response in COVID-19 patients is a significant area of research. We developed a 3D model of these SAg-like regions and determined their interaction pattern with TCR. A proper understanding of hyper inflammation in severe COVID-19 cases may help solve the mystery of the death of these patients (García, 2020; Tay et al., 2020). Our study allows us to illustrate the TCR and downstream regulatory pathways for the hyperinflammatory response in COVID-19 patients. Our MSA analysis found another partial super SAg-like region (ANQFNSAIGKI) from the S-glycoprotein of SARS-CoV-2 in the Wuhan strain and B.1.351, B.1.1.28/triple mutant, B.1.1.7, and B.1.429 variants.

The study of the insight into the antigenicity of SARS-CoV-2 variants has fundamental importance. It helps us illustrate the dynamic interaction between SARS-CoV-2 variants with humans or other hosts and also allows us to understand the immunopathogenesis of SARS-CoV-2 variants. Understanding the antigenic variations has immense importance. Antigenic variation might be a significant factor in comparing the antigenicity among the virus variants. Antigenic variation can help us to understand the virus fitness. At the same time, it can also explain how a virus can reinfect hosts by escaping the immune memory. Antigenic variations, diversification, conservation, and the total number of antigens in a particular virus protein need to be studied. However, antigenic variations have been studied in different viruses from time to time (Zost et al., 2019). The antigenic variations have also been studied in all human coronaviruses and SARS-CoV-2 (Kumar et al., 2020). Researchers have also investigated antigenic variations in spike protein in SARS-CoV-2 (Harvey et al., 2021). Some researchers have tried to illustrate the antigenic variations in SARS-CoV-2 variants (Mittal et al., 2022).

However, the total number of epitopes in a spike protein might be one criterion to determine the antigenicity comparing the SARS-COV-2 and SARS-CoV (Zheng and Song, 2020). Here we compared the total number of CTL epitopes in variants of SARS-CoV-2. Our study showed that B.1.1.7 (Alpha) of spike protein displayed the highest number of CTL epitopes compared to Wuhan strain, B.1.351 (Beta), B.1.1.28/triple mutant (P.1), and B.1.429 (epsilon). However, the surface accessibility of those epitopes is also a significant factor in interacting nAb (neutralizing antibody), and a need of further study for these variants. At the same time, there is also a need to understand how antigenic epitopes are presented to the T cell by MHC (major histocompatibility complex) molecules (both class I and class II molecules; Forni et al., 2021).

## Limitations of the study

The study has performed a comprehensive analysis of the CTL epitopes or SAg-like region of the total S-glycoprotein of the Wuhan strain, B.1.351, B.1.1.28/triple mutant, B.1.1.7, and B.1.429 variants and predicted the antigenicity through computational biology. The analyses were performed in various directions. Every data accumulated through the bioinformatics methods for Wuhan strain and emerging mutant variants is essential for future researchers and society. The arrival of emerging variants during the pandemic made the pandemic period more critical. At this point of urgency, our data predicting the potent antigenicity through bioinformatics tools which is very significant and highly beneficial for society. However, data acquired by us needs further validation through *in vitro* and *in vivo* methods, which is a limitation of this study. Therefore, we urgently urge future researchers to validate our data to end the pandemic crisis and prepare for a future pandemic.

## Conclusion

Several studies have focused on the identification and characterization of immunogenic CD8^+^ T cell epitopes. MHC-I molecules naturally contain 8-aa to 11-aa length peptide chains, which is the main consequence of the proteasomal degradation of antigens. This antigen is derived from infection or self-peptides (Blum et al., 2013). Our 9-mer CTL epitopic study helped identify the primary mechanism of antigen processing of the S-glycoprotein of SARS-CoV-2 *via* the MHC-I molecules of CD8^+^ T cells.

This study initiated several questions regarding the antigen processing of the S-glycoprotein of this virus *via* MHC-I molecules for CD8^+^ T cells. The researcher should solve these questions. We should also know about the antigen processing of the S-glycoprotein of the Wuhan strain and other emerging VOCs/VOIs such as B.1.351, B.1.1.28/triple mutant, B.1.1.7, and B.1.429 variants. At the same time, it is also necessary to understand more about the peptide–MHC-I complexes formation for the Wuhan strain and other emerging VOCs/VOIs such as B.1.351, B.1.1.28/triple mutant, B.1.1.7, and B.1.429 variants.

Different scientists have developed immunogenicity models for CTL epitopes (Harndahl et al., 2012; Calis et al., 2013). However, specific immunogenicity models for CTL epitopes of SARS-CoV-2 S-glycoprotein remain to be developed.

We identified the highest virulent epitopes with a significant mutation within the S-glycoprotein of SARS-CoV-2 emerging VOCs/VOIs (B.1.351, B.1.1.28/triple mutant, B.1.1.7, and B.1.429 variants) and the Wuhan strain. Our results showed that the mutations might be responsible for the high antigenicity identified in the B.1.1.7 variant. This variant might help activate more CD8^+ ^ T cells *via* the MHC-I pathway than in the South African, Brazilian, and Wuhan variants. It might help activate more CD4^+^ T cells *via* the MHC-II pathway and cytokine signaling. In the future, the molecular mechanism by which CD8^+^ T cells distinguish antigens from immunogenic property antigens of S-glycoprotein of wild strain and their variants should be evaluated.

## Data availablity statement

The original contributions presented in the study are included in the article/Supplementary material, further inquiries can be directed to the corresponding authors.

## Author contributions

CC: conceptualization, data acquisition, and writing–original draft. MB and ARS: formal analysis, validation, and reviewing and editing. BM and S-SL: formal analysis and validation. CC and E-MS: supervision and funding. All authors contributed to the article and approved the submitted version.

## Funding

This study was supported by the Hallym University Research Fund and the Basic Science Research Program through the National Research Foundation of Korea (NRF), funded by the Ministry of Education (NRF-2020R1C1C1008694 and NRF-2020R1I1A3074575).

## Conflict of interest

The authors declare that the research was conducted in the absence of any commercial or financial relationships that could be construed as a potential conflict of interest.

## Publisher’s note

All claims expressed in this article are solely those of the authors and do not necessarily represent those of their affiliated organizations, or those of the publisher, the editors and the reviewers. Any product that may be evaluated in this article, or claim that may be made by its manufacturer, is not guaranteed or endorsed by the publisher.

## Supplementary material

The Supplementary material for this article can be found online at: https://www.frontiersin.org/articles/10.3389/fmicb.2022.895695/full#supplementary-material

SUPPLEMENTARY FIGURE S1 Cluster formation of 15 mer CTL epitopes of S-glycoprotein of Wuhan strain and B.1.351, B.1.1.28/triple mutant, B.1.1.7, and B.1.429 variant using the threshold 10% level to 80% level. **(A)** Cluster formation at 10% level. **(B)** Cluster formation at 20% level. **(C)** Cluster formation at 30% level. **(D)** Cluster formation at 40% level. **(E)** Cluster formation at 50% level. **(F)** Cluster formation at 60% level. **(G)** Cluster formation at 70% level. **(H)** Cluster formation at 80% level.

SUPPLEMENTARY FIGURE S2 Cluster formation of 20 mer CTL epitopes of S-glycoprotein of Wuhan strain and B.1.351, B.1.1.28/triple mutant, B.1.1.7, and B.1.429 variant using the threshold 10% level to 80% level. **(A)** Cluster formation at 10% level. **(B)** Cluster formation at 20% level. **(C)** Cluster formation at 30% level. **(D)** Cluster formation at 40% level. **(E)** Cluster formation at 50% level. **(F)** Cluster formation at 60% level. **(G)** Cluster formation at 70% level. **(H)** Cluster formation at 80% level.

SUPPLEMENTARY FIGURE S3 Percentage of secondary structure component (α-helix and β-sheet) calculated from CD spectra of S-glycoprotein of the Wuhan strain and B.1.351, B.1.1.28/triple mutant, B.1.1.7, B.1.429 variants. **(A)** Percentage of α-helix and β-sheet of S-glycoprotein of the Wuhan variant. **(B)** Percentage of α-helix and β-sheet of S-glycoprotein of the B.1.351 variant. **(C)** Percentage of α-helix and β-sheet of S-glycoprotein of the B.1.1.28/triple mutant variant. **(D)** Percentage of α-helix and β-sheet of S-glycoprotein of the B.1.1.7 variant. **(E)** Percentage of α-helix and β-sheet of S-glycoprotein of the B.1.429 variant.

SUPPLEMENTARY FIGURE S4 Comparison of α-helix and β-sheet and β turns of S-glycoprotein of the Wuhan strain and B.1.351, B.1.1.28/triple mutant, B.1.1.7, B.1.429 variants. **(A)** Comparison of α-helix and β-sheet of S-glycoprotein among Wuhan strain and B.1.351, B.1.1.28/triple mutant, B.1.1.7, B.1.429 variants. **(B)** Comparison of β turns of S-glycoprotein among Wuhan strain and B.1.351, B.1.1.28/triple mutant, B.1.1.7, B.1.429 variants.

## Abbreviations

SARS-CoV-2: Severe acute respiratory syndrome coronavirus 2
COVID-19: Coronavirus disease 2019
RBD: Receptor-binding domain
S-protein: Spike glycoprotein
CTL: Cytotoxic T lymphocytes
CD: Circular dichroism
TCR: T-cell antigen receptor
VOC: Variants of concern.
VOI: Variants of interest
MSA: Multiple sequence alignment
SAg: Superantigen
RMSD: Root-mean-square deviation
MHC: Major histocompatibility complex
HLA: Human leukocyte antigens
PDB: Protein Data Bank
HMMs: Hidden Markov models
