# Supplementary figures and images for: B.1.1.7 (Alpha) variant is the most antigenic compared to Wuhan strain, B.1.351, B.1.1.28/triple mutant and B.1.429 variants
Source: Front Microbiol. 2022 Aug 12;13:895695. doi: 10.3389/fmicb.2022.895695 (PMC9411949; doi:10.3389/fmicb.2022.895695)

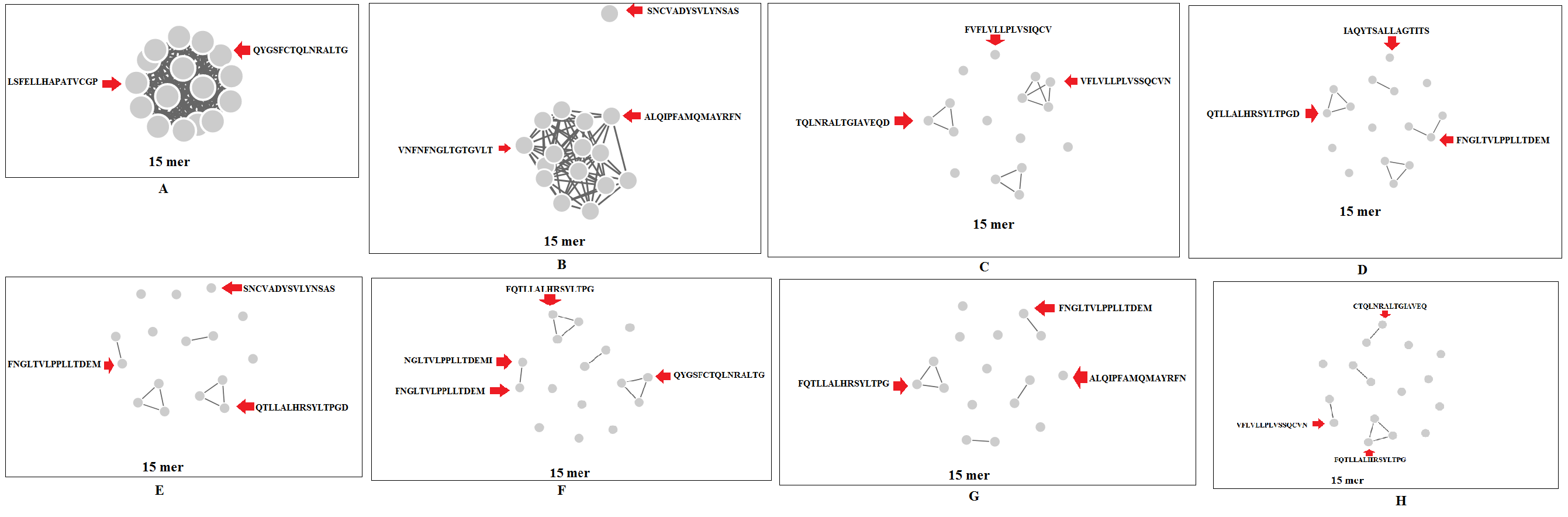

Supplement: SUPPLEMENTARY FIGURE S1 — Cluster formation of 15 mer CTL epitopes of S-glycoprotein of Wuhan strain and B.1.351, B.1.1.28/triple mutant, B.1.1.7, and B.1.429 variant using the threshold 10% level to 80% level. (A) Cluster formation at 10% level. (B) Cluster formation at 20% level. (C) Cluster formation at 30% level. (D) Cluster formation at 40% level. (E) Cluster formation at 50% level. (F) Cluster formation at 60% level. (G) Cluster formation at 70% level. (H) Cluster formation at 80% level. [file Image_1.TIF]

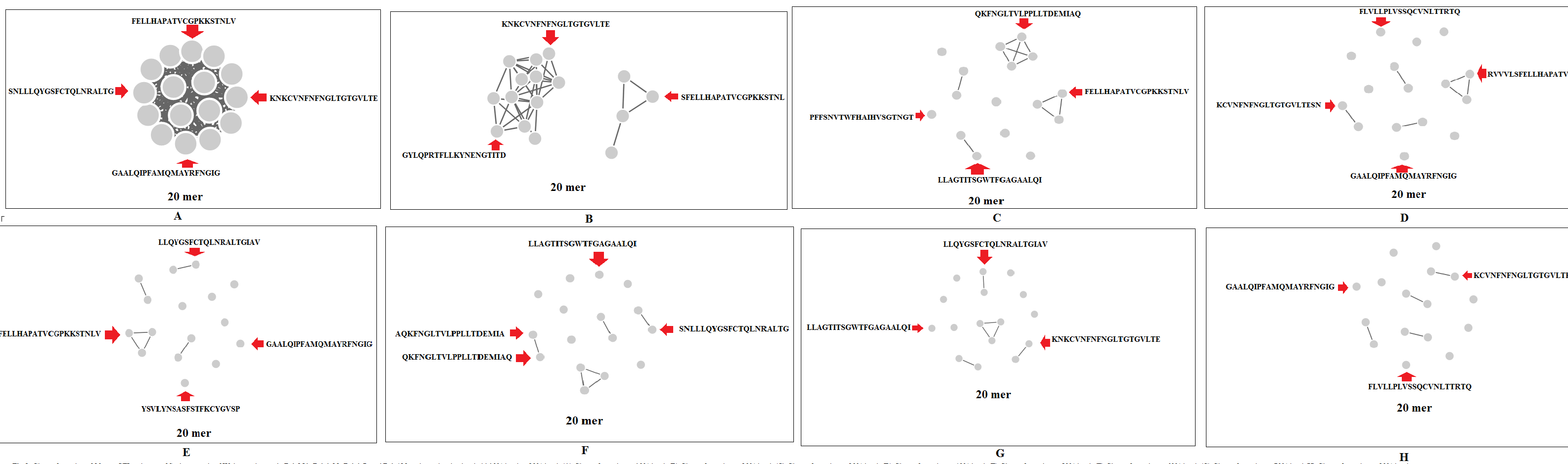

Supplement: SUPPLEMENTARY FIGURE S2 — Cluster formation of 20 mer CTL epitopes of S-glycoprotein of Wuhan strain and B.1.351, B.1.1.28/triple mutant, B.1.1.7, and B.1.429 variant using the threshold 10% level to 80% level. (A) Cluster formation at 10% level. (B) Cluster formation at 20% level. (C) Cluster formation at 30% level. (D) Cluster formation at 40% level. (E) Cluster formation at 50% level. (F) Cluster formation at 60% level. (G) Cluster formation at 70% level. (H) Cluster formation at 80% level. [file Image_2.TIF]

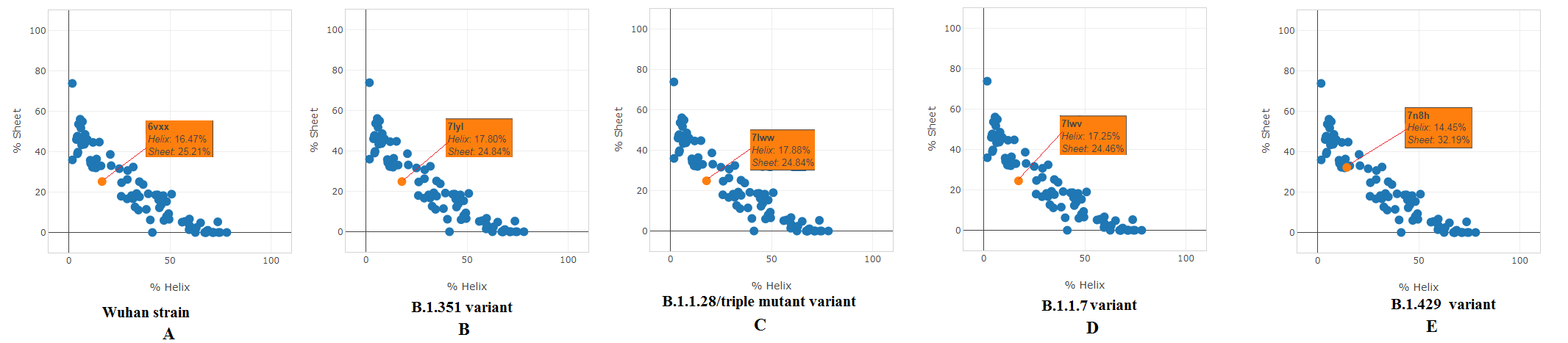

Supplement: SUPPLEMENTARY FIGURE S3 — Percentage of secondary structure component (α-helix and β-sheet) calculated from CD spectra of S-glycoprotein of the Wuhan strain and B.1.351, B.1.1.28/triple mutant, B.1.1.7, B.1.429 variants. (A) Percentage of α-helix and β-sheet of S-glycoprotein of the Wuhan variant. (B) Percentage of α-helix and β-sheet of S-glycoprotein of the B.1.351 variant. (C) Percentage of α-helix and β-sheet of S-glycoprotein of the B.1.1.28/triple mutant variant. (D) Percentage of α-helix and β-sheet of S-glycoprotein of the B.1.1.7 variant. (E) Percentage of α-helix and β-sheet of S-glycoprotein of the B.1.429 variant. [file Image_3.TIF]

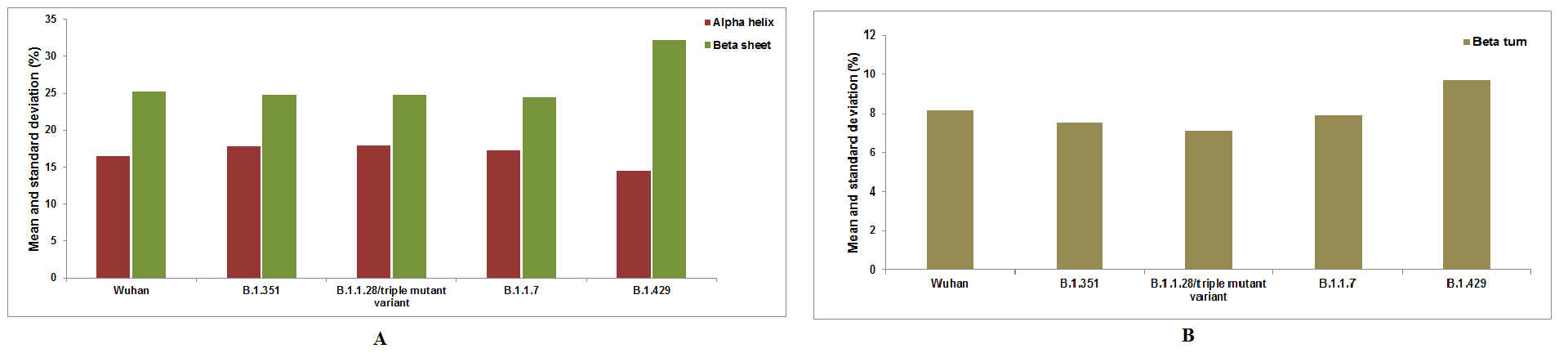

Supplement: SUPPLEMENTARY FIGURE S4 — Comparison of α-helix and β-sheet and β turns of S-glycoprotein of the Wuhan strain and B.1.351, B.1.1.28/triple mutant, B.1.1.7, B.1.429 variants. (A) Comparison of α-helix and β-sheet of S-glycoprotein among Wuhan strain and B.1.351, B.1.1.28/triple mutant, B.1.1.7, B.1.429 variants. (B) Comparison of β turns of S-glycoprotein among Wuhan strain and B.1.351, B.1.1.28/triple mutant, B.1.1.7, B.1.429 variants. [file Image_4.TIF]
