# Supplementary material for: B.1.1.7 (Alpha) variant is the most antigenic compared to Wuhan strain, B.1.351, B.1.1.28/triple mutant and B.1.429 variants
Source: Front Microbiol. 2022 Aug 12;13:895695. doi: 10.3389/fmicb.2022.895695 (PMC9411949; doi:10.3389/fmicb.2022.895695)
Supplement: Supplementary file 5 [file Table_1.DOCX]

| **Variants name** | **Epitopes** | **Epitopes position** | | **VaxiJen Score** |
| --- | --- | --- | --- | --- |
|  |  | **S-protein domain** | **S-protein chain** |  |
| B.1.351 | FANPVLPFN | NTD | 79-87 | 0.2328  (Non-antigen) |
|  | GLPQGFSAL | NTD | 215-223 | 0.1868  (Non-antigen) |
|  | NIADYNYKL | RBD | 417-425 | 1.5325  (Antigenic) |
|  | KGFNCYFPL | RBD | 484-492 | 0.5711  (Antigenic) |
|  | VENSVAYSN | SD2 | 701-709 | 0.4171  (Antigenic) |
| B.1.1.28/triple mutant | SQCVNLTTR | NTD | 13-21 | 1.5476  (Antigenic) |
|  | SPGSASSVA | SD1 | 680-688 | 0.4280  (Antigenic) |
|  | FQPTYGVGY | RBD | 497-505 | 0.4935  (Antigenic) |
|  | NIADYNYKL | RBD | 417-425 | 1.0536  (Antigenic) |
| B.1.1.7 | EILDITPCS | SD1 | 583-591 | 0.4935  (Antigenic) |
|  | FQPTYGVGY | RBD | 497-505 | 1.5485  (Antigenic) |
|  | EGFNCYFPL | RBD | 481-489 | 0.5453  (Antigenic) |
|  | RDIDDTTDA | SD1 | 564-572 | 0.7902  (Antigenic) |
|  | FTISVTTEI | SD2 | 715-723 | 0.8535  (Antigenic) |
|  | IITTHNTFV | CD | 1111-1119 | 0.4551  (Antigenic) |
| B.1.429 | CMESEFRVY | NTD | 152-160 | 0.4587  (Antigenic) |
|  | RYRLFRKSN | RBD | 452-460 | -0.6465  (Non-antigen) |
|  | FQFCNDPFL | NTD | 132-140 | -0.2493  (Non-antigen) |
| Wuhan | VVFLHVTYV | HR2 | 1060-1068 | 1.5122  (Antigenic) |
|  | GKQGNFKNL | NTD | 181-189 | 1.0607  (Antigenic) |
|  | GIYQTSNFR | NTD | 311-319 | 0.5380  (Antigenic) |
|  | VSPTKLNDL | RBD | 382-390 | 1.4610  (Antigenic) |
|  | FKNHTSPDV | CD | 1156-1164 | 0.4846  (Antigenic) |

**Table 1.** Identification and comparative analysis of CTL epitopes of Wuhan strain and B.1.351, B.1.1.28/triple mutant, B.1.1.7, B.1.429 variant.
