# Supplementary material for: B.1.1.7 (Alpha) variant is the most antigenic compared to Wuhan strain, B.1.351, B.1.1.28/triple mutant and B.1.429 variants
Source: Front Microbiol. 2022 Aug 12;13:895695. doi: 10.3389/fmicb.2022.895695 (PMC9411949; doi:10.3389/fmicb.2022.895695)
Supplement: Supplementary file 6 [file Table_2.DOCX]

| **Variant** | **Epitopes** | **Position** | **VaxiJen Score (Non-antigen/ antigen)** |
| --- | --- | --- | --- |
| Wuhan | IAQYTSALLAGTITS | 870-884 | 0.4188 (Probable NON-ANTIGEN) |
|  | DLFLPFFSNVTWFHA | 53-67 | 0.2472 (Probable NON-ANTIGEN) |
|  | QYGSFCTQLNRALTG | 755-769 | 0.7858 (Probable ANTIGEN) |
| B.1.351 | FNGLTVLPPLLTDEM | 855-869 | 0.2617 (Probable NON-ANTIGEN) |
|  | VNFNFNGLTGTGVLT | 539-553 | 1.2439 (Probable ANTIGEN) |
|  | CTQLNRALTGIAVEQ | 760-774 | 0.7454 (Probable ANTIGEN) |
|  | QTLLALHRSYLTPGD | 239- 253 | 0.6708 (Probable ANTIGEN) |
| B.1.1.28 | NGLTVLPPLLTDEMI | 856-870 | 0.1852 (Probable NON-ANTIGEN) |
|  | FQTLLALHRSYLTPG | 238-252 | 0.5789 (Probable ANTIGEN) |
|  | VFLVLLPLVSSQCVN | 3-17 | 0.5954 (Probable ANTIGEN) |
| B.1.1.7 | LSFELLHAPATVCGP | 510-524 | 0.5062 (Probable ANTIGEN) |
|  | TRFQTLLALHRSYLT | 233-247 | 0.3262 (Probable NON-ANTIGEN) |
|  | ALQIPFAMQMAYRFN | 890-904 | 1.0112 (Probable ANTIGEN) |
|  | TQLNRALTGIAVEQD | 758-772 | 0.4153 (Probable ANTIGEN) |
|  | SNCVADYSVLYNSAS | 356-370 | -0.1317 (Probable NON-ANTIGEN) |
| B.1.429 | LSFELLHAPATVCGP | 513-527 | 0.5062 (Probable ANTIGEN) |
|  | FVFLVLLPLVSIQCV | 2-16 | 1.1570 (Probable ANTIGEN) |
|  | VNFNFNGLTGTGVLT | 539-553 | 1.2439 (Probable ANTIGEN) |
|  | CTQLNRALTGIAVEQ | 760-774 | 0.7454 (Probable ANTIGEN) |

**Supplementary table S1:** Identification and comparative analysis and of 15 mer CTL epitopes of Wuhan strain

and B.1.351, B.1.1.28/triple mutant, B.1.1.7, B.1.429 variant

| **Variant**  **Supplementary table S2:** Identification and comparative analysis and of 20 mer CTL epitopes of Wuhan strain and B.1.351, B.1.1.28/triple mutant, B.1.1.7, B.1.429 variant | **Epitopes** | **Position** | **VaxiJen Score (Non-antigen/ antigen)** |
| --- | --- | --- | --- |
| Wuhan | KNKCVNFNFNGLTGTGVLTE | 535-554 | 1.2763 (Probable ANTIGEN) |
|  | LLAGTITSGWTFGAGAALQI | 877-896 | 0.3990 (Probable NON-ANTIGEN) |
|  | LLQYGSFCTQLNRALTGIAV | 753-772 | 0.7445 (Probable ANTIGEN) |
| B.1.351 | YSVLYNSASFSTFKCYGVSP | 365-384 | 0.5100 (Probable ANTIGEN) |
|  | GYLQPRTFLLKYNENGTITD | 268-287 | 0.5657 (Probable ANTIGEN) |
|  | PFFSNVTWFHAIHVSGTNGT | 57-76 | 0.6969 (Probable ANTIGEN) |
|  | SNLLLQYGSFCTQLNRALTG | 750-769 | 0.5992 (Probable ANTIGEN) |
| B.1.1.28 | FLVLLPLVSSQCVNLTTRTQ | 4-23 | 1.0702 (Probable ANTIGEN) |
|  | KCVNFNFNGLTGTGVLTESN | 537-556 | 1.2355 (Probable ANTIGEN) |
|  | ITGRLQSLQTYVTQQLIRAA | 997-1016 | 0.0539 (Probable NON-ANTIGEN) |
| B.1.1.7 | FELLHAPATVCGPKKSTNLV | 512-531 | 0.3229 (Probable NON-ANTIGEN) |
|  | AQKFNGLTVLPPLLTDEMIA | 849-868 | 0.2120(Probable NON-ANTIGEN) |
|  | GAALQIPFAMQMAYRFNGIG | 888-907 | 0.9065 (Probable ANTIGEN) |
| B.1.429 | RVVVLSFELLHAPATVCGPK | 509-528 | 0.3924 (Probable NON-ANTIGEN) |
|  | SFELLHAPATVCGPKKSTNL | 514-533 | 0.4040 (Probable ANTIGEN) |
|  | QKFNGLTVLPPLLTDEMIAQ | 853-872 | 0.1990 (Probable NON-ANTIGEN) |

**Supplementary Table S3:** Comparative experiment of CD spectra generation and its RMSD calculation using Wuhan strain and B.1.351, B.1.1.28/triple mutant, B.1.1.7, B.1.429 variant. The experiment uses different parameters for comparative experiments.

| **Sl. no** | **Experimental parameters** | **B.1.351** | **B.1.1.28/**  **triple mutant variant** | **B.1.1.7** | **B.1.429** |
| --- | --- | --- | --- | --- | --- |
|  | Exp. Filename | CD sectra_7LYL.txt | CD sectra_7LWW.txt | CD sectra_7LWV.txt | CD sectra_7N8H.txt |
|  | No. Members | 1 | 1 | 1 | 1 |
|  | Max RMSD threshold | 0.12 | 0.19 | 0.15 | 0.45 |
|  | Subset Mean Spectra RMSD | 0.114 | 0.166 | 0.133 | 0.440 |
|  | Subset RMSD Std. Dev. | +/- 0.000 | +/- 0.000 | +/- 0.000 | +/- 0.000 |
|  | Closest Prediction to Exp. | pdb6vxx.ent | pdb6vxx.ent | pdb6vxx.ent | pdb6vxx.ent |
|  | Closest Prediction RMSD | 0.114 | 0.166 | 0.133 | 0.440 |
|  | Furthest Prediction to Exp. | pdb6vxx.ent | pdb6vxx.ent | pdb6vxx.ent | pdb6vxx.ent |
|  | Furthest Prediction RMSD | 0.114 | 0.166 | 0.133 | 0.440 |

**Supplementary Table S4:** Secondary structure component of Wuhan strain and B.1.351, B.1.1.28/triple mutant, B.1.1.7, B.1.429 variant

| **Sl. no** | **Secondary structure type** | **Mean and standard deviation (%)** | | | | |
| --- | --- | --- | --- | --- | --- | --- |
|  |  | **Wuhan strain** | **B.1.351** | **B.1.1.28/**  **triple mutant variant** | **B.1.1.7** | **B.1.429** |
|  | Helix 1 | 14.75 +/- 0.0 | 15.6 +/- 0.0 | 15.73 +/- 0.0 | 15.51 +/- 0.0 | 11.33 +/- 0.0 |
|  | Helix 2 | 1.72 +/- 0.0 | 2.2 +/- 0.0 | 2.15 +/- 0.0 | 1.74 +/- 0.0 | 3.12 +/- 0.0 |
|  | Antiparallel Sheet 1 | 14.33 +/- 0.0 | 14.09 +/- 0.0 | 13.93 +/- 0.0 | 13.86 +/- 0.0 | 19.71 +/- 0.0 |
|  | Antiparallel Sheet 2 | 8.14 +/- 0.0 | 8.06 +/- 0.0 | 8.52 +/- 0.0 | 8.52 +/- 0.0 | 9.36 +/- 0.0 |
|  | Parallel Sheet | 2.73 +/- 0.0 | 2.69 +/- 0.0 | 2.38 +/- 0.0 | 2.07 +/- 0.0 | 3.12 +/- 0.0 |
|  | Turn | 8.12 +/- 0.0 | 7.5 +/- 0.0 | 7.12 +/- 0.0 | 7.91 +/- 0.0 | 9.71 +/- 0.0 |
|  | Other | 50.21 +/- 0.0 | 49.86 +/- 0.0 | 50.17 +/- 0.0 | 50.39 +/- 0.0 | 43.65 +/- 0.0 |
